# Pitfalls and Inherent Biases in Liquid Handling Robotics: Investigations in Automation for SI Traceable Measurements

**DOI:** 10.1021/acs.analchem.6c00078

**Published:** 2026-05-26

**Authors:** Tabatha Hambidge, Steven Corless, Simon Cowen, Michael Short, Chris Hopley, Patrick Sears

**Affiliations:** † NML, 25781 LGC, The Priestley Centre, Guildford GU2 7XY, U.K.; ‡ School of Chemistry & Chemical Engineering, 3660 University of Surrey, Guildford GU2 7XH, U.K.

## Abstract

Industries such as pharmaceuticals, petrochemicals, and clinical research are adopting automated sample preparation processes to streamline repetitive tasks and improve efficiency. As robotics underpins more complex methods of analysis, the evaluation and optimization of workflows and apparatus are vital. In this study, the accuracy and precision of an automated workflow were investigated using two liquid handling platforms with syringes and integrated balances, with the focus on their suitability for accurate multianalyte quantification. The methodology was developed by optimizing key parameters such as the syringe draw and dispense speeds. Additionally, the physical setup was evaluated as the syringe tip, vial sizes, and decapper tool all played a vital role in the performance of the system. The performance of robotic sample preparation was compared to traditional gravimetric manual preparation of amino acid blends, using double exact matching isotope dilution mass spectrometry (DEM-IDMS). After optimal conditions for the liquid-handling robotics were established, the accuracy of the DEM-IDMS method with automated sample preparation was assessed using NIST SRM 2389a amino acids in 0.1 mol/L HCl. The optimization process successfully reduced a systematic bias identified in the robotic workflow from 10% to <3.5%. Moreover, the measurement uncertainty achieved with the optimized automated method (≤5.3%, expanded at the 95% confidence interval for all amino acids) was comparable to that of manual preparation, while saving a full day of analyst time (1 h setup versus 7 h manually weighing). The research outputs highlighted are invaluable to improve the understanding of the advantages and remaining challenges in the use of automated sample preparation for high-accuracy analysis.

## Graphical abstract

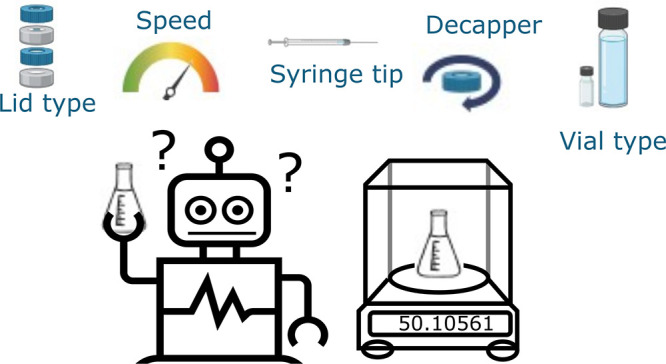

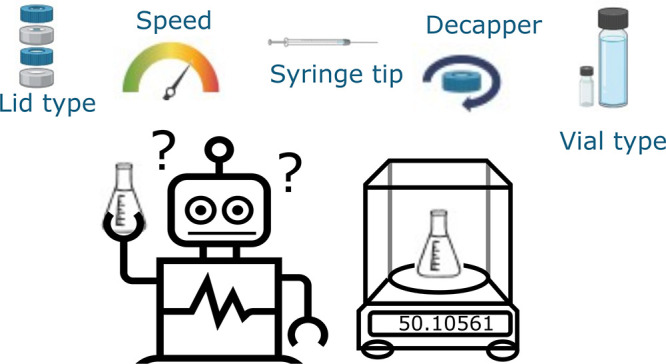

## Introduction

Automated liquid handlers have rapidly appeared in the modern laboratory, carrying out repetitive liquid additions with claims of greater accuracy, precision, and efficiency compared with manual handling.^1,2^ Examples of automated sample preparation include solid-phase extraction^3,4^ and serial dilution steps for calibrations.^5,6^ In life sciences and bioanalysis, automation can be found in screening and high-throughput laboratories to improve productivity and reduce error.^7^

Despite their widespread adoption, the metrological accuracy of these systems, especially in terms of SI-traceable quantification and measurement uncertainty, is underexplored. The performance of these systems needs to be rigorously assessed. Traditional evaluations often compare volumetric and gravimetric additions, measuring recovery for accuracy or assessing precision by repeating experiments.^8–11^ While these approaches provide useful baseline performance metrics, they do not always capture the full analytical impact of automated workflows.

Laboratories have identified subtle errors in liquid handlers, highlighting the need for system validation.^12^ Studies have shown how different automated systems, when evaluated based on overall analytical output rather than isolated liquid-handling steps, can influence observed results including introducing analyte-specific biases.^2^ Other work has also shown how environmental factors (temperature, humidity, and pressure) and instrument parameters (sampling speed, air gaps, etc.) impact the performance of automated pipetting systems.^13^

Poor precision, poor accuracy, and unknown sources of bias can have significant consequences for analytical methods, particularly in real-world applications where decisions rely on reliable data. In regulated industries such as food and pharmaceuticals, even small deviations in measurement accuracy can lead to significant impacts, including misclassification of product quality, failure to detect contaminants, or incorrect potency assessments. These errors can have implications for patient safety, regulatory compliance, product recalls, and economic loss.^14–17^ Likewise, a limited understanding of true measurement uncertainty, especially when automation is introduced, can have tangible impacts, including patient-safety risks in pharmaceutical and clinical settings where biased results may influence clinical or regulatory decisions.^18^ For trace level analysis, even minor variability in sample preparation can disproportionately affect quantification accuracy and detection reliability.^19^ These challenges are becoming increasingly important as physical automation technologies are combined with machine learning and artificial intelligence-driven optimization, creating systems whose performance depends on robust analytical measurements.^20^ Robust metrological validation of automated workflows is essential to ensuring data integrity and maintaining confidence in analytical decisions.

Prep and load (PAL)-based autosamplers, developed by CTC Analytics, have long been used for automated sample introduction in chromatographic systems, and several aspects of their performance have been investigated. For example, the influence of vial closure systems on gas chromatography (GC)-based analysis is well-established. Septa materials have been shown to introduce volatile contaminants, contributing to background signals and bias in GC measurements.^21,22^ Similarly, the integrity of vial sealing has been identified as a critical factor in headspace analysis, where septum puncture and elevated internal pressures can lead to gas leakage and reduced analytical accuracy and repeatability.^23^

In more recent years, PAL-based and other injection systems have evolved beyond simple injection platforms to enable online sample preparation workflows, including dilution, derivatization, standard addition, and other preparative steps.^24,25^ While such capabilities significantly expand their application space, they also introduce additional sources of variability that have not yet been fully understood from a quantitative perspective.

However, automated sample preparation systems have been reported to achieve repeatability comparable to, or in some cases better than, manual workflows. One study found their automated method achieved 3–5% relative standard deviation (RSD) and within commonly accepted limits such as those described by the Horwitz equation.^26^ Meanwhile, another study showed accuracy ranges of 70–120% with RSD values below 10% for automated workflows.^27^ However, these evaluations are often based on method-level performance metrics and do not explicitly address traceability, bias, or the contribution of automated liquid handling to the overall measurement uncertainty. As a result, the quantitative reliability of automated sample preparation systems remains insufficiently understood, particularly in applications that require high metrological confidence.

Double exact matching isotope dilution mass spectrometry (IDMS) (DEM-IDMS) is a technique that assigns mass fractions to the International System of Units (SI) of the kilogram with a full uncertainty statement.^28^ Although very accurate, it is a laborious and iterative process for a highly skilled analyst, which involves gravimetrically preparing, diluting, and combining the calibration blend, sample blend, and labeled spike multiple times. Utilizing this workflow provides the opportunity to use a high-order reference method to assess the accuracy of the robotic system with any bias identified and an estimated uncertainty budget. This study leverages DEM-IDMS not as a target method but as a benchmark to evaluate the metrological integrity of syringe-based robotic systems in multianalyte quantification.

Previous work has explored automation of single IDMS by using highly precise robotic preparation for plutonium analysis.^29^ However, the study focused on the use of automated pipettes to measure a single analyte, with the procedure conducted within a glovebox to eliminate human interaction with the sample. The automation strategy primarily optimized the sequence order to minimize evaporation, since no lids were used, and optimized stirring for sample homogenization.^29^ To the authors’ knowledge, no studies have yet evaluated automated sample preparation workflows against manual methods for the high-accuracy quantification of several amino acids using DEM-IDMS, or involving robotic systems equipped with syringes.

Here, for the first time, we propose a workflow based on DEM-IDMS to evaluate the use of syringe-based robotic systems for automated sample preparation and high-accuracy quantification of key organic compounds. The workflow has been designed to assess the performance of the robotic systems by comparing the assigned mass fraction values obtained through automation with those of a certified reference material (CRM) manually prepared and commercially available from the National Institute of Standards and Technology (NIST). To achieve this, single AA CRMs were used as a model system to optimize and validate the automated based methodology. A standard comparison was performed using NIST SRM 2389a (amino acids in 0.1 mol/L HCl), serving as the benchmark for manual preparation. Two syringe-based liquid-handling robots equipped with integrated balances were evaluated for their ability to perform this multianalyte gravimetric workflow. These robotic systems were chosen because of their compatibility with a balance for gravimetric preparation. This study was used as an illustrative example and provides critical insights into the reproducibility and traceability of automated workflows, offering a foundation for the future development of SI-traceable automated reference methods.

## Materials and Methods

### Analytes and Reagents

l-Leucine (CRM 6012-a), l-alanine (CRM 6011-a), l-valine (CRM 6015-a), l-lysine monohydrochloride (CRM 6018-a), l-isoleucine (CRM 6013-a), l-proline (CRM 6016-a), l-arginine (CRM 6017-b), l-phenylalanine (CRM 6014-a), l-tyrosine (CRM 6019-a), and l-methionine (CRM 6023-a) were purchased from the National Metrology Institute of Japan (NMIJ; Tsukuba, Japan). SRM 2389a amino acids in 0.1 mol/L HCl was purchased from the National Institute of Standards and Technology (NIST; Maryland, USA) and is certified for all the amino acids listed above.

Stable isotopes l-leucine (^13^C_6_,^15^N), l-alanine (^13^C_3_,^15^N), l-valine (^13^C_5_,^15^N), l-lysine·2HCl (^13^C_6_,^15^N_2_), l-isoleucine (^13^C_6_), l-proline (^13^C_5_,^15^N), l-arginine·HCl (^13^C_6_), l-phenylalanine (^13^C_9_,^15^N), l-tyrosine (^13^C_9_,^15^N), and l-methionine (methyl-^13^C,D_3_) were supplied by Cambridge Isotopes Laboratories (Massachusetts, USA).

18 MΩ·cm ultrapure water was prepared in-house using an Elga water purifier system. Ultrapure hydrochloric acid and LC–MS grade acetonitrile were purchased from Romil, (Cambridge, UK). MTBSTFA (*N*-*tert*-butyldimethylsilyl-*N*-methyltrifluoroacetamide) with 1% *tert*-butyldimethylchlorosilane (TBDMSCl) was supplied by Merck (Dorset, UK).

### Standard Preparation

#### DEM-IDMS Methodology

Double exact matching IDMS (DEM-IDMS; a primary ratio method) was used to value assign the mass fraction of multiple analytes (amino acids) in solution with high accuracy and low uncertainty, traceable to the SI.^28^ Individual primary AA CRMs with SI traceable certified values and uncertainties were used to prepare the individual stocks for the robot to prepare mixed solutions from. Additionally, the robot-prepared solutions were compared to a manual mixed AA CRM (NIST SRM 2389a amino acids) with known traceable values and uncertainties. The DEM-IDMS process and uncertainty calculation are detailed in Supporting Information 1.

#### Solution Preparation

The process of gravimetric preparation of AA stocks, dilutions, and blends in 0.1 molar hydrochloric acid by an analyst or automated liquid handlers are shown in Figure 1. For the manual preparation, the NIST SRM 2389 mixed AA material was diluted 10-fold. For the automated blend preparation, individual stocks were prepared at 1 mg/g. Mixed dilutions of natural and labeled amino acids were prepared to exactly match the NIST CRM 2389 mixed AA material mass fractions after the 10-fold dilution—these values are shown in Supporting Information 2.

**Figure 1:**
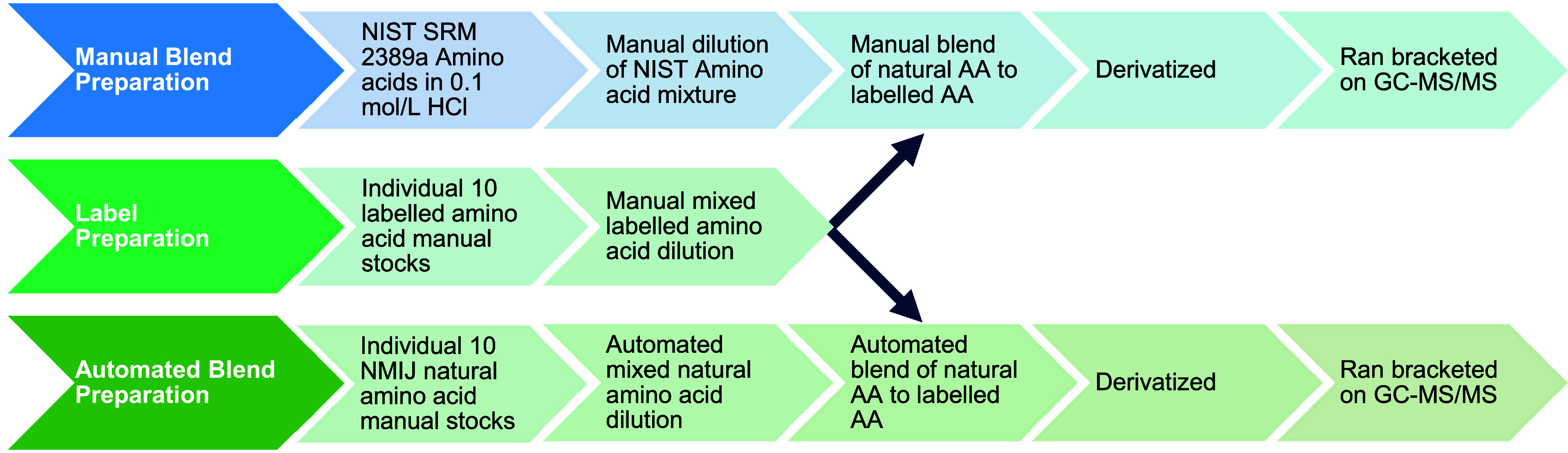
Schematic of standard preparation comparison by DEM-IDMS.

#### Manual Preparation

Manual dilutions and blends were conducted using an annually calibrated Mettler XP205 five-figure balance and Gilson positive displacement pipettes into glass vials. The lids were uncapped and recapped during liquid transfers, and the pipet tips were changed before each transfer.

#### 7696A Agilent Workbench

The 7696A Agilent workbench (California, USA) is a liquid handling sample preparation robot that has a built-in, annually calibrated, Mettler micro balance with a deionizer. It contains a vortex mixer and 50 spaces for 2 mL autosampler vials which can be heated or chilled. The workbench has two turrets with syringes that can be changed to different sizes (10 μL—1 mL) and includes solvent wash, sample wash, and sample pump options. The system is software-controlled (G4529AA), which allows for method building, resource tracking, and sequence operation.

#### GERSTEL Multipurpose Sampler (MPS)

The Gerstel MPS modular system (Mülheim, Germany) was configured with a five-figure Sartorius balance (with integrated antistatic tool); two syringes that could be changed to different sizes (10 μL—5 mL); a 1 mL pipet tool; trays for 2 mL, 4 mL, 10 mL, and 20 mL vials; a vortex mixer; two decappers; fast wash; and a solvent reservoir. The system is software-controlled, which allows for method building and sequence running (Maestro Version 1.5.7.12).

#### Derivatization

Both manual and robotic prepared solutions were derivatized (manually) to improve gas chromatography tandem mass spectrometry (GC–MS/MS) performance. 100 μL of the blend solutions was lyophilized in a vacuum centrifuge for 1 h 30 min at 40 °C, then reconstituted in 100 μL of derivatizing agent (MTBSTFA with 1% TBDMSCl), and the vials heated at 85 °C in a shaking oven for 3 h before being analyzed via GC–MS/MS.

### GC–MS/MS Method

Both an Agilent 7010B GC–MS/MS with CTC PAL RSI 120 and a Shimadzu TQ8040 NX GC–MS/MS with an AOC-6000 Plus autosampler were used for this analysis, with either Restek RXi-5HT 30 m × 0.25 mm, 0.25 μm, or Agilent HP-5MS UI (same dimensions) columns.

The same method was used for all samples. 1 μL of sample was injected using a 1:10 split ratio with a 300 °C inlet temperature. The flow rate was 1.2 mL/min with an oven temperature program starting at a 130 °C hold for 3 min, ramped at a rate of 10 °C/min to 240 °C, followed by a second ramp of 35 °C/min to 320 °C with a final hold for 3 min. The operating parameters were as follows: positive electron ionization, transfer line temperature 300 °C, source temperature 230 °C, and a gain factor of 20. Compound-specific multiple reaction monitoring channels (MRMs) were set up to monitor for the derivatized analytes and their fragments with multiple transitions for each analyte, which can all be found listed in Supporting Information 3.

### Statistical Analysis

Statistical comparisons were performed using a range of tests selected according to the characteristics of each data set. Student’s *t* tests, *F*-tests, and linear regression were applied where appropriate to assess differences between groups. All analyses were conducted using Microsoft Excel, and statistical significance with 95% confidence was defined as *p* < 0.05. Complete data sets and corresponding statistical outputs are provided throughout the Supporting Information.

## Results and Discussion

### Method Development and Optimization

Preliminary observations during method development suggested greater than expected discrepancies when comparing the mass fractions of amino acids and their associated uncertainties in model samples, measured using DEM-IDMS, between manual DEM-IDMS blend preparations and those made using automated robotic systems. These observations prompted a systematic investigation into the potential sources of bias, including parameters such as pump speeds, wash protocols, sequence order, and air gap settings as well as the physical setup such as syringe tips, vial lids, decapper tool, and vial sizes. The results of these optimizations are detailed in the sections below.

#### Initial Configurations and Considerations

##### System Checks

The performance of the system was checked before use, using a simple check weight experiment (see the Supporting Information 4 for more information).

##### Lids

The Agilent Workbench transfers standard autosampler vials using a gripper tool, and initial tests were conducted to determine whether preslit/slit and screw cap/crimp cap lids contributed to evaporation. In these experiments, the robot dispensed 100 mg of water into empty vials by piercing the septum with a syringe, after which the vials were reweighed at multiple time points over 72 h. Any reduction in weight was attributed to evaporation, with results presented in Supporting Information 5.

After 72 h, cumulative loss was consistently greater in preslit caps (ca. −1.2 μL) compared to nonslit caps (ca. −0.4 μL), across both screw and crimp formats, for both water and acetonitrile. Evaporation was more pronounced in the acetonitrile experiments (ca. −18 μL and −4 μL). Linear regression analysis of evaporation trends revealed strong significant differences between preslit and nonslit crimp caps for both solvents (*p* = 8.7 × 10^–9^ and 4.7 × 10^–11^). For screw caps, a significant difference was observed between preslit and nonslit vials in acetonitrile (*p* = 5.9 × 10^–7^) but not in water (*p* = 0.08), likely due to the lower overall evaporation of water. In the water experiments, screw nonslit caps differed significantly from crimp nonslit caps (*p* = 0.008), whereas screw preslit and crimp preslit caps did not (*p* = 0.07). The opposite pattern was observed in acetonitrile (screw nonslit/crimp nonslit *p* = 0.19 and screw preslit/crimp preslit *p* = 0.01.)

Overall, nonslit lids were found to be the superior option for minimizing evaporation, while differences between screw and crimp caps were minimal.

The Gerstel MPS system transports vials using a magnet using Gerstel-specific vials. These vials were fitted with screw caps and did not feature preslit options.

##### Optimizing Settings and Parameters

Methods were developed so that results from automated systems (using a syringe) would replicate those of manual preparation by an analyst using a pipette. The settings and parameters (listed in Table 1) were optimized through visual assessment to eliminate errors and sources of contamination.

**Table 1:** Settings and Parameter Considerations from Initial Method Development on the Agilent and Gerstel Automated Liquid Handling Systems

important settings and considerations	Agilent Workbench	Gerstel MPS
washes	the method can be programmed to wash the syringe with solvent before and after every addition	the method can be programmed to wash the syringe with the solvent before and after every addition
	the wash volume can be changed	the wash volume can be changed
	up to two solvents can be used—4 mL × 10 vials	up to two solvents can be used—any size bottle can be used
	it is useful to do this every time to stop cross-contamination	it is useful to do this every time to stop cross-contamination
dispense wash	the method can be programmed to conduct a dispense wash within the add step—it picks up some sample and puts it to waste	a dispense wash must be programmed as its own step
	it is useful to do this to precondition the syringe needle	it is useful to do this to precondition the syringe needle
dispense pumps	on both systems, the methods can be programmed to conduct a dispense pump within the add step—it fills and dispenses sample within the syringe. The volume and number of pumps can be adjusted. This mimics what the analyst does with a pipette and helps to condition the syringe needle and prevent air bubbles	
speeds	on both systems, the draw and dispense speeds can be adjusted within the add step for each syringe size. Slow draw prevents air bubbles, fast dispense reduces droplets forming at the end of the syringe	
viscosity delay	a viscosity delay can be set within the add step	a viscosity delay can be set within the add step
		a postadd delay can also be programmed in seconds on the Gerstel system to wait before moving the syringe
air gap	the syringe can be programmed to pull an air gap after pulling up sample or solvent, within the vial, to prevent it from dripping or leaking as the syringe moves to another vial. Set as a percent within the add step	the syringe cannot be programmed to pull an air gap—when trying to add this as a separate step, the arm would reset to the rail and would not pull an airgap within the vial
in vial vacuum	if the vials are overfilled, there is a vacuum effect, so only 1000 μL can be added to 1200 μL vials. The lids also cannot be overtightened for the same reason, and the septa must be level and not obstructed	if the vials are overfilled there is an in-vial vacuum effect if piercing the septum. Each vial has a different capacity. If the vial is decapped then this issue is eliminated
syringe limits	the workbench cannot reliably take 400 μL with a 500 μL syringe (it needs to be ∼65% full)	the Gerstel does not accurately work within 10% of the syringe capacity (900 μL in 1 mL syringe)
overfill	the syringe can be programmed to overfill after pulling up the sample or solvent, within the vial, to make the addition more accurate. The overfill is sent to waste after the addition. Set as a percent within the add step settings	the syringe can be programmed to overfill after pulling up the sample or solvent, within the vial, to make the addition more accurate. The overfill is sent to waste after the addition. This is known as “accurate add” and is set as a volume (minimum of 10%) within the add step settings

##### Using Weight to Check the Accuracy of Dispensing

Using weight to verify that the system was dispensing, the correct volumes proved to be a reliable indicator of well-optimized system settings. Following optimization, the correct volumes were dispensed consistently and repeatably. In a series of 12 consecutive water additions (between 25 and 4100 μL), an error of less than 0.5% for any single measurement was observed on both robots using multiple syringes, the data set is shown in Supporting Information 6. Setting information will also be supplied in Supporting Information 6.

#### Troubleshooting for Dilution Steps

Once correctly configured, DEM-IDMS experiments were conducted on an Agilent Workbench. The first experiment involved blending, where equal amounts of natural and labeled amino acids were added to a vial at their working mass fractions. These blends were compared to those prepared by an analyst. Following optimization of the workflow utilizing the findings in Table 1, the robot-prepared blends were acceptable for two consecutive blending experiments, each with six replicate blends.

The second experiment consisted of a dilution from the stock solution to the working solution introduced before blending. This approach was inconsistent, showing a bias of up to 10% when compared to dilutions and blends prepared by the analyst. It was hypothesized that the inconsistency could be due to the different volumes required for the dilutions, with the bias potentially arising when the volumes are not equal, although the gravimetric measurement should account for any volume discrepancies. Nonetheless, the measured value differed from the gravimetrically calculated value, suggesting a more fundamental issue affecting the process.

To determine whether the error was related to the limited vial size and flexibility of the Agilent Workbench, the same experiments were repeated by using the Gerstel MPS robot. This system has an analytical balance that can be used manually or integrated with automation, and the system can use a variety of vial sizes; therefore, the process mirrors manual preparation by an analyst more closely. Both automated systems use a syringe, piercing a vial septum to transfer the liquid. The Gerstel system allowed both one-step and two-step dilution experiments, unlike the Agilent, which required a two-step dilution (because of vial volume constraints). Both the blending-only and dilution experiments were performed on each system, with results presented for phenylalanine in Figure 2. The graph shows the gravimetric values with an expanded uncertainty range (pink shaded area), the average of the three measured mass fractions from the replicates (red line), and the individual measured mass fractions from each replicate (blue). The uncertainties for the value calculated from the gravimetric measurements and the average value measured by mass spectrometry should overlap in the absence of bias.

**Figure 2:**
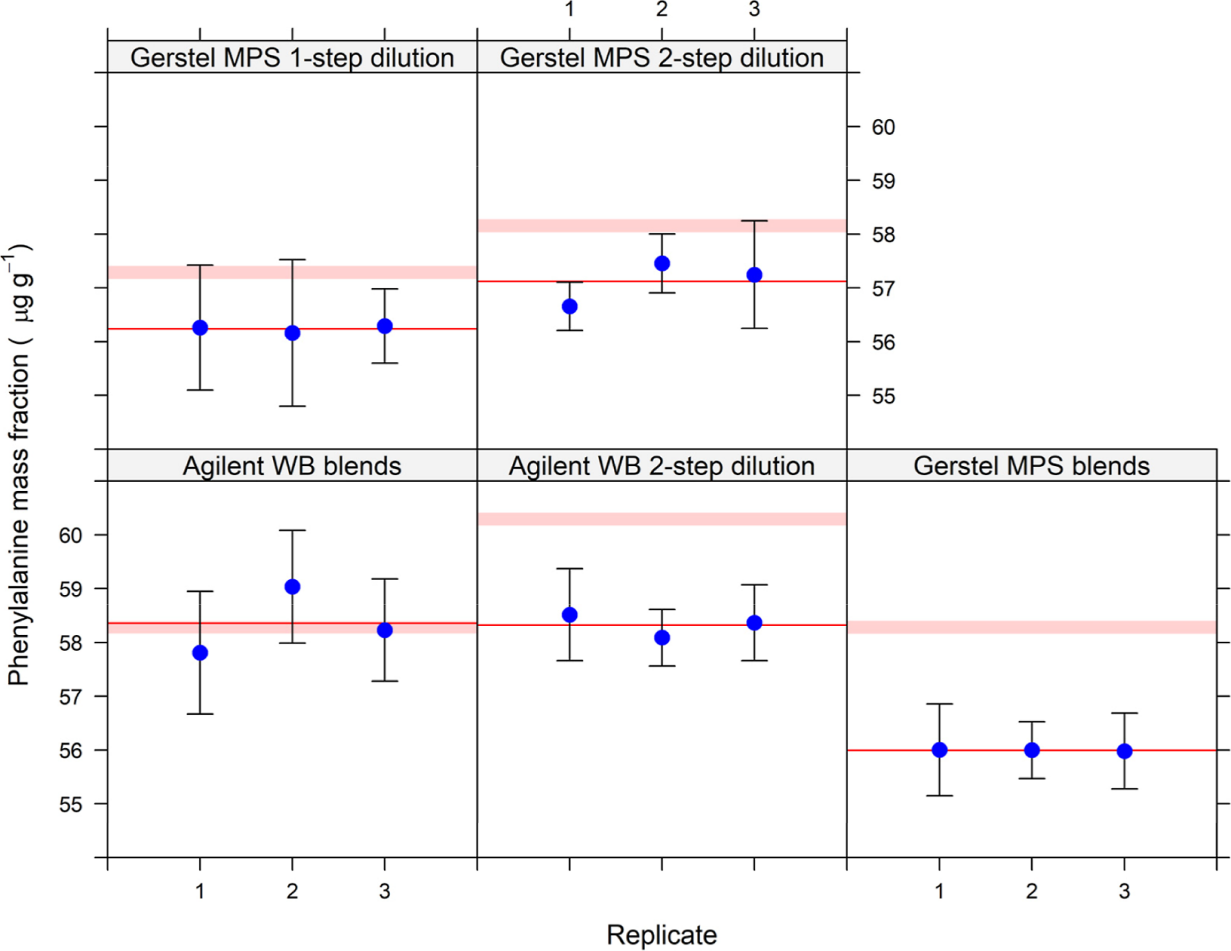
Graph of phenylalanine measured mass fractions from each three-way comparison experiment replicate (blue) versus the average of the three (red line) and the gravimetric value with expanded uncertainty range (pink shaded area)—error bars display ± the expanded uncertainty (*k* = 2). The pink gravimetric value should overlap uncertainties for a comparable experiment.

The measured values with the Agilent system agreed for the blending-only experiment, but not for the two-step dilution. The Gerstel system produced consistent responses for the three blend replicates, but the gravimetric values varied from the average measured values for the blends and two-step dilution up to 5%. The data obtained from the one-step dilution performed on the Gerstel did overlap uncertainties. Student’s *t* tests were conducted between the average measured value and the gravimetric value (shown in Supporting Information 7), these results indicate significant differences for the Agilent workbench two-step dilution (*p* = 1.16 × 10^–2^), and the Gerstel MPS blends (*p* = 8.56 × 10^–3^). Both systems were inconsistent, and the measured value was often lower than the gravimetric value, suggesting an unaccounted-for error or bias with both preparation robots. This underestimation has important implications for quantitative accuracy, as it may lead to consistent under-reporting of analyte concentrations and reduced comparability with gravimetrically prepared standards if it was not identified. Such bias could compromise data reliability, particularly in applications requiring high quantitative accuracy, and may limit the suitability of these systems for trace-level or regulatory analyses without further optimization or correction.

The common components between the robots that differed from manual preparation were the use of a syringe and piercing of the septum. This experiment was replicated manually using a syringe and a Mettler XP205 balance. The experiment was initially conducted through the septum; the experiment was then repeated by opening the lid between transfers (removing the septum piercing step). Results from this manual experiment are available in Supporting Information 8. These data show that the septum-piercing methods produced variable results (with one of the three measurements having a significant difference between the gravimetric and measured value), while the lid-removal method consistently produced acceptable results (no significant difference between gravimetric and measured value). It was concluded from the observation of the experiment that one source of inconsistent responses and bias stemmed from residue left on the septum, which affected the weight but not the mass fraction.

Additionally, during this experiment, it was noted that the 500 μL syringe could not create an air gap after picking up solvent or samples as expected. An air gap is when the sample is drawn into the syringe, the syringe tip is moved above the surface of the liquid, and a small amount of air is drawn into the syringe. Ideally, when the air gap is pulled, the air stays at the bottom of the syringe, preventing the liquid from dripping out of the syringe. Nevertheless, in the 500 μL syringe, likely due to its surface area, the air bubble floated to the top of the syringe, and therefore, there was no plug of air preventing drips from the syringe, and this may contribute to droplet formation on the syringe’s tip. When the air gap was tested on a 250 μL syringe and a 100 μL syringe, the air plug stayed at the bottom of the syringe. This is an important consideration when aiming to prevent the residue from being left of the septum.

#### Syringe Considerations

Syringes can be purchased with blunt, cone, or bevelled tips, and the tip shape can influence the formation of droplets. Investigation determined that the 100 μL blunt tip Gerstel syringe consistently formed droplets, regardless of the syringe program and dispense speed. Conversely, the 250 μL cone tip did not always form solvent droplets especially after program optimization of the dispense speed; this is illustrated in Figure 3.

**Figure 3:**
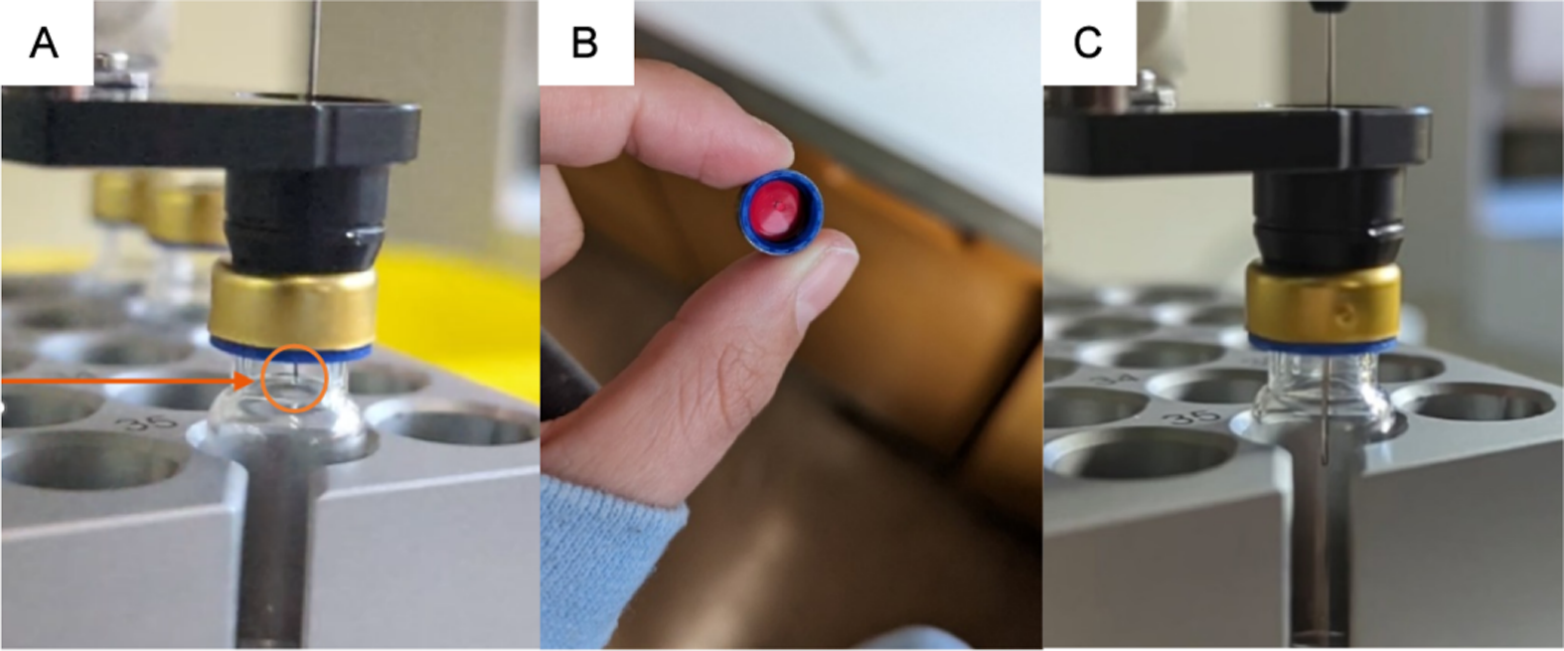
Pictures of droplet formation. (A) Shows a droplet forming when using the 100 μL blunt tip syringe. (B) Shows the droplet being caught in the septum. (C) Shows the lack of a droplet when optimizing speeds on a 250 μL cone ended tip; however, this result was variable. Pictures (A,C) are stills from videos filming at 8× slow speed.

Droplet formation was also affected by the distance that the syringe needle protruded into the vial. If it did not protrude enough, then the surface tension and an electrostatic attraction would cause a droplet to form on the inner surface of the lid of the vial. If conducting an addition without a lid, the droplet would stay attached to the syringe housing, and no liquid would be transferred into the vial. Subsurface addition was considered; however, back-exchange could bias gravimetric and measured values.

#### Evaporation

Having identified the septum as a potential source of bias and a major source of variability, evaporation tests were performed during decapping. 500 mg of water was placed in a 10 mL vial, which was then repeatedly decapped, left open for 2 min to replicate automated handling conditions (the average time taken for the system to perform the required additions), and then recapped without any additional material added. The vial was weighed after each decapping/recapping event, for simplicity, all mass-to-volume calculations assumed water density of 1 g/cm^3^. This experiment was repeated by piercing the vial for a comparison.

In addition, a control experiment with three analysts decapping and recapping vials without adding any volume was also performed. They each had the vial open for approximately 20 s while simulating their normal manual processes. The difference in wait time between the manual and automated methods represents the normal operating duration of each workflow. All three sets of data are presented in Figure 4.

**Figure 4:**
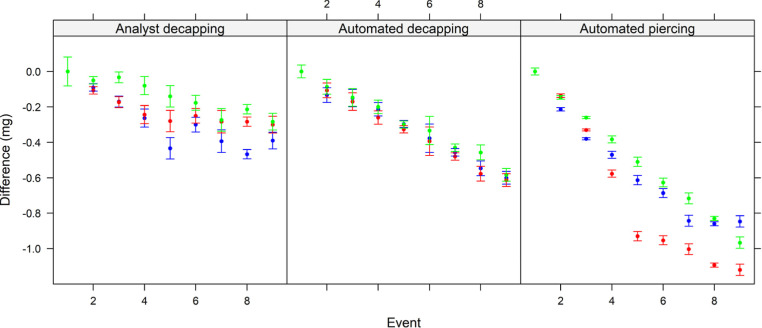
Comparison of evaporation over three methods—analyst decapping, automated decapping, and automated piercing. Each color represents an analytical replicate. The cumulative loss of water after each piercing or decapping event. Error bars are equal to ± the standard deviation of weighing replicates (*n* = 3). For simplicity, all mass to volume calculations assumed a water density of 1 g/cm^3^.

The cumulative losses were compared across the three experiments. Automated piercing resulted in the greatest loss, followed by automated decapping and finally manual decapping (0.98 mg, 0.60 mg, and 0.32 mg, respectively). Multiple *t* tests confirmed that the differences in total loss between all three methods were statistically significant (*p* = 0.009, 0.002, and 0.001).

To assess variability, the standard deviation of the evaporation curves was calculated, taking into account the cumulative loss over eight events. This was done by calculating the average variance at each event (Supporting Information 9). Automated piercing showed the highest variability, manual decapping was intermediate, and automated decap/recap exhibited the lowest variability (standard deviation of trend of curve, respectively: 0.138, 0.092, and 0.031). A one-tailed *F*-test revealed significant differences between manual and automated decapping (*p* = 3.2 × 10^–4^), as well as between automated decapping and automated piercing (*p* = 5.6 × 10^–6^). However, the difference between automated piercing and manual decapping was not significant (*p* = 0.056) (Supporting Information 9).

An additional comparison was conducted between two evaporation experiments in acetonitrile: automated piercing and automated decapping (Supporting Information 10). Total cumulative evaporation was significantly greater with automated decapping than with piercing (−5.74 mg vs −0.76 mg). In contrast, the standard deviation of the evaporation curve was markedly higher in the piercing experiment compared to decapping (0.85 vs 0.19). This increased variability in the piercing data was partly attributable to a step change observed in one replicate, likely caused by a fragment of the septum being removed.

In summary, automated decapping proved to be the most consistent method, exhibiting the lowest variability. However, it also resulted in substantial evaporation, which must be taken into consideration.

A number of parameters were optimized to minimize the time the vials were decapped on the Gerstel system, including increasing the draw and dispense speeds, reducing the number of syringe washes and syringe fill pumps, and adjusting the sequence of syringe cleaning so that the syringe would be cleaned before the vial was uncapped and washed again after the vial was recapped (this required programming additional steps outside of the “addition” step).

The headspace volume of the vial was another factor that could influence evaporation, and so the experiment was repeated (Figure 5) but with 100 mg, 500 mg, or 2000 mg of water initially added into the 10 mL vial. Each evaporation experiment was performed on the same day to reduce the variation in the ambient temperature and humidity in the room.

**Figure 5:**
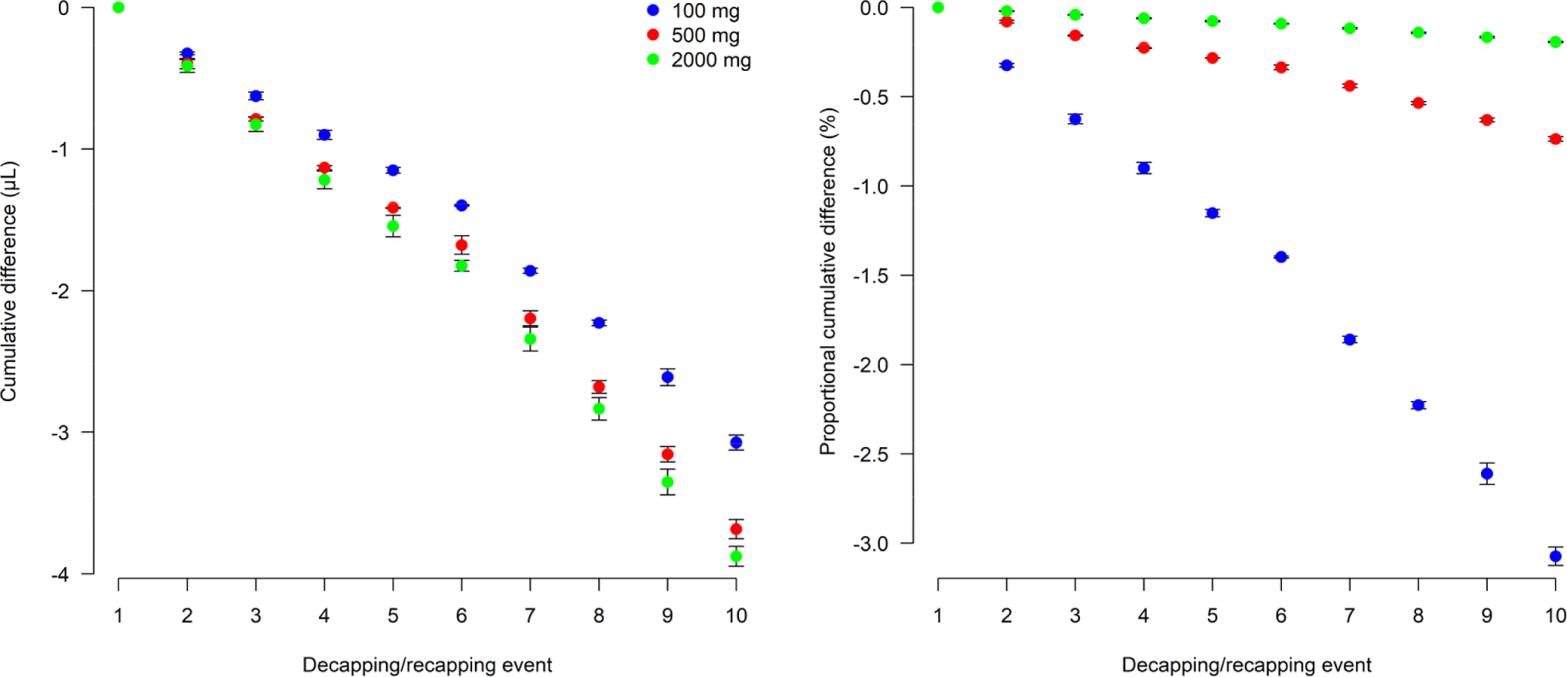
Robot decapping evaporation experiment for different volumes of water from a 10 mL vial after the lid is opened, wait for 2 min, and closed again. Cumulative differences are shown on the left and percentage differences are shown on the right. Error bars are equal to ± the standard deviation of analytical replicates (*n* = 3). For simplicity, all mass to volume calculations assumed a water density of 1 g/cm^3^.

The trend in the evaporation looked similar upon initial inspection of the graphs, although the proportional cumulative loss increased with the greater headspace volume in the vial (total loss of 0.19% from the 2000 mg solution, 0.74% evaporated from the 500 mg solution, and 3.07% from the 100 mg solution). To evaluate whether the rate of change differed significantly between groups, we examined the relationship between the mass change and event occurrence. Because this relationship appeared to follow an exponential or power law form, we attempted to linearize the data and found that when both axes were log-transformed, the data was linearized, meaning it is consistent with a power law relationship. This transformation allowed us to test differences in power relationship (slopes) and the rate of change (intercepts) across groups and volumes in both the absolute and proportional data (Supporting Information 11).

The resulting straight-line fits confirmed that the underlying relationship was exponential or power-nature. Statistical tests indicated that while the power relationships were not significantly different between groups for either the absolute or proportional data, the rate of change was for all of the proportional data and that the 2000 mg and 500 mg were statistically different from the 100 mg absolute data, but not from each other (Supporting Information 11). This suggests that the overall rate of change varied significantly between groups based on the starting volume even though the proportional steepness of the relationship remained consistent.

These large differences in the rate of change in the proportional data likely reflect limiting factors, such as surface area. A possible way to avoid this large proportional loss would be to add the bulk of the solution before the addition of the smaller stock solutions. These data also highlight the importance of selecting the correct vial size.

### Methodology Accuracy Assessment by Analysis of NIST SRM 2389a Amino Acids in 0.1 mol/L HCl

After further optimization of the sequence based on the earlier findings and inclusion of decapping, the Gerstel robot prepared a mixed ten AA calibrator whose value assigned the National Institute of Standards and Technology (NIST) standard reference material 2389a material successfully (Figure 6). All the values calculated by using the robotic workflow showed no significant difference to the certified value of the NIST material (*t*-test in Supporting Information 12), and therefore, no systematic bias was observed. The uncertainty of each measured value prepared by the liquid handler was ≤5.3%, which is comparable to an analyst.

**Figure 6:**
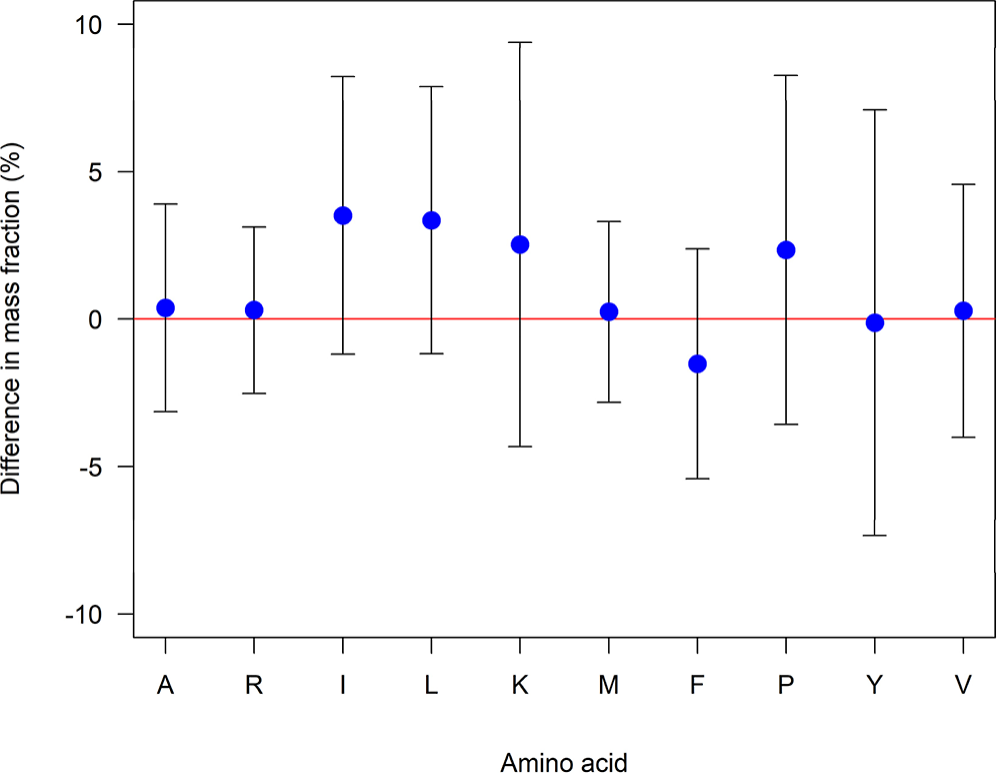
Certified value of the National Institute of Standards and Technology (NIST) material (red line) is compared to the measured value and associated uncertainty prepared by using the robotic liquid handling with the decapper tool (blue). The black error bars are equal to ± the expanded uncertainty of the difference (95% coverage factor corresponding to the number of degrees of freedom) relative to the NIST value. The robot prepared measured value overlap with the certified value for every analyte meaning a successful comparison with no statistical difference between the measured and certified value.

As a comparison, this experiment was also completed using syringes and piercing the septum. These data gave biased results as six of the ten AA measured values were statistically different from the NIST value, and all measured results were greater than the certified values (Supporting Information 13).

Although the automated preparation required approximately the same total elapsed time as manual preparation (around 7 h), the analyst’s active involvement was limited to the initial setup of the robot. Once configured, the system operated unattended, effectively saving the analyst an entire working day of manual effort.

This successful comparison between the automated workflow and NIST-certified metrological standards demonstrates that automated systems can operate accurately, precisely, and traceably, with an acceptably low uncertainty. Improved understanding and control of evaporation, droplet formation, gravimetric accuracy, and overall system setup are particularly critical for achieving reliable results, and these experiments provide a foundation for evaluating additional solvent systems and analytes at lower detection limits. Furthermore, metrologically valid performance data from automated liquid-handling systems can support future integration with machine-learning optimization strategies.

## Conclusions

This research contributes to the broader adoption of automated liquid handling in analytical chemistry and biochemistry by improving the understanding of the challenges in robotic performance and by providing a metrologically rigorous evaluation of syringe-based robotic systems. By applying DEM-IDMS to evaluate syringe-based robotic systems for the first time for the accurate quantification of amino acids, this study has highlighted several unexpected sources of bias and inaccuracy in the automated gravimetric preparation of solutions. Syringe droplet residues were shown to affect the accuracy of the solutions produced when using automated systems. Through minimization of droplets via optimization of draw and dispense speeds, correct syringe selection, and utilization of a decapping tool, we have reduced the observed bias, demonstrating that accurate automated gravimetric workflows are feasible. The expanded uncertainty for each preparation was calculated as ≤5.3% which is comparable to manual preparation by a skilled analyst, demonstrating that high-accuracy automated workflows are suitable for SI-traceable value assignment with associated uncertainties that meet calibration requirements.

While this study focused primarily on aqueous systems, preliminary investigations of acetonitrile evaporation highlighted solvent-specific challenges. Future work should extend this metrological framework to include a broader range of organic solvents and analyte classes, enabling a more comprehensive evaluation of automation applicability across diverse chemical environments.

Beyond technical validation, this work offers valuable insights into the operational limits of automated gravimetric solution and sample preparation, which can be applied as a powerful cost and time-saving strategy across a multitude of industries. In a real-world setting, adoption of this strategy could save an entire day of a highly skilled analyst’s time and retain low measurement uncertainty. While the pitfalls of automation highlighted here can impact accuracy, these findings demonstrate how they can be overcome to achieve robust implementation in any laboratory setting, from research and development through to quality assurance and quality control.

## Supplementary Material



## Notes

DEM-IDMS and uncertainty calculations; mass-fraction comparison with NIST SRM 2389a; multiple reaction monitoring channels (MRMs); system checks; lid-type evaporation experiments; data and settings for accurate dispensing; Student’s *t*-test comparison of Agilent and Gerstel systems; manual syringe experiment data; additional water-evaporation data; acetonitrile-evaporation data; linear-regression data for evaporation across volumes; comparison with the NIST material by decapping; and comparison with the NIST material by piercing (DOCX)

## References

[1] Kong F., Yuan L., Zheng Y. F., Chen W. (2012). Automatic liquid handling
for life science: A critical review of the current state of the art. J. Lab. Autom..

[2] Yang Y. K., Reichman H. A., Bankhead C. D., McNamee J. A. (2021). Evaluate
the comparability
of two automated liquid handling systems for clinical toxicology assays. Drug Test. Anal..

[3] León-González Z., Ferreiro-Vera C., Priego-Capote F., Luque de Castro M. D. (2011). Targeting
metabolomics analysis of the sunscreen agent 2-ethylhexyl 4-(N,N-dimethylamino)­benzoate
in human urine by automated on-line solid-phase extraction-liquid
chromatography-tandem mass spectrometry with liquid chromatography-time-of-flight/mass
spectrometry confirmation. J. Chromatogr. A.

[4] Calderón-Santiago M., Priego-Capote F., Galache-Osuna J. G., Luque de Castro M. D. (2012). Determination
of essential amino acids in human serum by a targeting method based
on automated SPE–LC–MS/MS: Discrimination between artherosclerotic
patients. J. Pharm. Biomed. Anal..

[5] Patkin, A. J. Calibration Dilution Workflow for Automated Standard Preparation on the TurboMatrix MultiPrep+; Application Note 013990_01; PerkinElmer, 2018. Available: https://gcms.cz/labrulez-bucket-strapi-h3hsga3/4cfcf3091b914733815c2629eb7e7c0c/APP_Calibration_Dilution_Workflow_on_TurboMatrix_MultiPrep_013990_01.pdf [Online] (accessed March 09, 2022).

[6] Foster, F. D. ; Stuff, J. R. Automated Calibration Standard Preparation using the MAESTRO Software Calibration Standard Wizard; Application Note No. 224; GERSTEL, 2021. https://www.gerstelus.com/appnote/automated-calibration-standard-preparation-using-the-maestro-software-calibration-standard-wizard/.

[7] Fleischer H., Drews R. R., Janson J., Chinna Patlolla B. R., Chu X., Klos M., Thurow K. (2016). Application
of a Dual-Arm Robot in
Complex Sample Preparation and Measurement Processes. J. Lab. Autom..

[8] Amino Acid Analysis using Andrew+ Automated Preparation—Aug 19 2022—Danielle Cullen, Niamh Stafford, Leanne Davey, Norma Breen, Steven Calciano Ning Zhang—Life Science News Articles—Labmate Online. Available: https://www.labmate-online.com/article/laboratory-products/3/waters-corporation/amino-acid-analysis-using-andrew-automated-preparation/3187 [Online] (accessed Sept 26, 2022).

[9] Taylor P. B., Ashman S., Baddeley S. M., Bartram S. L., Battle C. D., Bond B. C., Clements Y. M., Gaul N. J., McAllister W. E., Mostacero J. A. (2002). A Standard Operating Procedure for Assessing
Liquid Handler Performance in High-Throughput Screening. J. Biomol. Screen..

[10] Bessemans L. (2016). Automated Gravimetric Calibration to Optimize
the Accuracy and Precision
of TECAN Freedom EVO Liquid Handler. J. Lab.
Autom..

[11] Geersing T. H., Klous M. G., Franssen E. J. F., van den Heuvel J. J. G., Crul M. (2020). Robotic compounding
versus manual compounding of chemotherapy:
Comparing dosing accuracy and precision. Eur.
J. Pharm. Sci..

[12] Britt-Rodriquez K., Daniel J., Hayden J. (2024). Surreptitious
pipetting errors on
a vendor-programmed liquid handler. J. Mass
Spectrom. Adv. Clin. Lab.

[13] Schuster J., Kamuju V., Zhou J., Mathaes R. (2024). Piston-driven automated
liquid handlers. SLAS Technol..

[14] Traple M. A. L., Saviano A. M., Francisco F. L., Lourenço F. R. (2014). Measurement
uncertainty in pharmaceutical analysis and its application. J. Pharm. Anal..

[15] Separovic L., Lourenço F. R. (2019). Measurement uncertainty and risk
of false conformity
decision in the performance evaluation of liquid chromatography analytical
procedures. J. Pharm. Biomed. Anal..

[16] Anagaw Y. K., Ayenew W., Limenh L. W., Geremew D. T., Worku M. C., Tessema T. A., Simegn W., Mitku M. L. (2024). Food adulteration:
Causes, risks, and detection techniques—review. SAGE Open Med..

[17] Soon J. M., Abdul Wahab I. R. (2021). Global
food recalls and alerts associated with labelling
errors and its contributory factors. Trends
Food Sci. Technol..

[18] Ellison, S. ; Williams, A. EURACHEM/CITAC Guide CG 4: Quantifying Uncertainty in Analytical Measurement, 3rd ed.; EURACHEM, 2012.

[19] Taverniers I., De Loose M., Van Bockstaele E. (2004). Trends in
quality in the analytical
laboratory. II. Analytical method validation and quality assurance. TrAC, Trends Anal. Chem..

[20] Rubinger L., Gazendam A., Ekhtiari S., Bhandari M. (2023). Machine learning
and
artificial intelligence in research and healthcare. Injury.

[21] Ulanowska A., Ligor T., Amann A., Buszewski B. (2012). Evaluation
of Septa Quality for Automatic SPME-GC-MS Trace Analysis. J. Chromatogr. Sci..

[22] Cooper, J. ; Cojocariu, C. ; Roberts, D. Non-Targeted Screening of Extractables from Vial Septa Using High-resolution Orbitrap GC-MS; Application Note 000206; ThermoScientific, 2021. Available: https://documents.thermofisher.com/TFS-Assets/CMD/Application-Notes/an-000206-gc-ms-extractables-vial-septa-an000206-na-en.pdf [Online] (accessed April 01, 2026).

[23] Xie W.-Q., Yu K.-X., Gong Y.-X. (2018). A double sealing technique for increasing
the precision of headspace-gas chromatographic analysis. J. Chromatogr. A.

[24] Whitecavage, J. A. ; Stuff, J. R. Automated Solid Phase Microextraction using the GERSTEL MPS Prepstation and MAESTRO Software; AppNote 2/2007, 2007. https://gcms.labrulez.com/labrulez-bucket-strapi-h3hsga3/2fa5c8d6546d41309cf2616f7828cf33/p-gc-an-2007-02.pdf.

[25] Agilent 7696A Sample Prep WorkBench: Quick Start Guide; G4529-90010; Agilent Technologies, 2021. Available: https://www.agilent.com/cs/library/usermanuals/public/G4529-90010.pdf [Online] (accessed April 02, 2026).

[26] Vorberg E., Fleischer H., Junginger S., Liu H., Stoll N., Thurow K. (2016). A Highly Flexible, Automated System Providing Reliable
Sample Preparation in Element- and Structure-Specific Measurements. J. Lab. Autom..

[27] Jeong W. T., Kim D., Lee S. H., Noh H. H. (2025). Automated
Matrix Dilution Injection
Method Utilizing Online Auto-Injection Program for Matrix-Matched
Calibration for the LC-MS/MS Analysis of Multiple Pesticide Residues. J. Sep. Sci..

[28] Henrion A. (1994). Reduction
of systematic errors in quantitative analysis by isotope dilution
mass spectrometry (IDMS): an iterative method. Fresenius’ J. Anal. Chem..

[29] Surugaya N., Hiyama T., Watahiki M. (2008). Automated Gravimetric Sample Pretreatment
Using an Industrial Robot for the High-Precision Determination of
Plutonium by Isotope Dilution Mass Spectrometry. Anal. Sci..

