# Supplementary material for: Pitfalls and Inherent Biases in Liquid Handling Robotics: Investigations in Automation for SI Traceable Measurements
Source: Anal Chem. 2026 May 26;98(22):16107–16. doi: 10.1021/acs.analchem.6c00078 (PMC13261617; doi:10.1021/acs.analchem.6c00078)
Supplement: Supplementary file 1 [file ac6c00078_si_001.docx]

**Supplementary information for:**

Pitfalls and inherent biases in liquid handling robotics: investigations in automation for SI traceable measurements

*Tabatha Hambidge* ^1,2^, Steven Corless^1^,* *Simon Cowen^1^, Michael Short^2^, Chris Hopley^,2^, Patrick Sears^2^*

*[tabatha.hambidge@lgcgroup.com](mailto:tabatha.hambidge@lgcgroup.com)*

*^1^ LGC, NML, The Priestley Centre, Guildford, GU2 7XY*

*^2^ School of Chemistry & Chemical Engineering, University of Surrey, Guildford, GU2 7XH*

**Contents**

[Supplementary Information 1 – DEM-IDMS and uncertainty calculations 2](#_Toc226388448)

[DEM-IDMS process 2](#_Toc226388449)

[Uncertainty calculation 3](#_Toc226388450)

[Supplementary Information 2 – Mass fractions when compared to NIST SRM 2389a 4](#_Toc226388451)

[Supplementary Information 3 – Multiple reaction monitoring channels (MRMs) 5](#_Toc226388452)

[Supplementary Information 4 – System checks 5](#_Toc226388453)

[Supplementary Information 5 – Lid-type evaporation experiments 6](#_Toc226388454)

[Supplementary Information 6 – Data and settings for accurate dispensing 8](#_Toc226388455)

[Agilent WorkBench workflow and settings for accurate dispensing 8](#_Toc226388456)

[Gerstel MPS workflow and settings for accurate dispensing 9](#_Toc226388457)

[Supplementary Information 7 – Student’s t-test information for Agilent and Gerstel comparison 10](#_Toc226388458)

[Supplementary Information 8 – Manual Syringe Experiment Data 12](#_Toc226388459)

[Supplementary Information 9 – Water Evaporation Experiment Additional Data 17](#_Toc226388460)

[Supplementary Information 10 – Acetonitrile Evaporation Experiment Data 20](#_Toc226388461)

[Supplementary Information 11 – Linear regression for evaporation from different volumes data 23](#_Toc226388462)

[Supplementary Information 12 – Comparison with NIST material by decapping 26](#_Toc226388463)

[Supplementary Information 13 – Comparison with NIST material by piercing 28](#_Toc226388464)

## Supplementary Information 1 – DEM-IDMS and uncertainty calculations

### DEM-IDMS process

When the measured ratio of the sample blend matches the measured ratio of the calibration blend, the solutions are exactly matched. When carrying out a standard comparison, the method works in the same way as measuring a sample. In this case, the robot-prepared standard was treated as the sample and the analyst prepared standard as the calibration blend (as shown in figure S1). The robot and analyst blend should ‘match’ with their calculated mass fraction with their gravimetric mass fraction by having overlapping uncertainties.

**Samples analysed by Mass Spectrometry Ratio of 1:1**

**R**


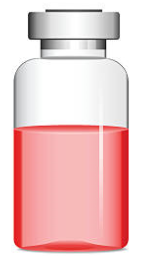


**Analyst prepped natural standard**


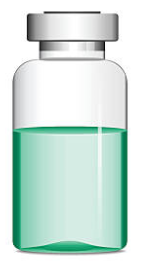

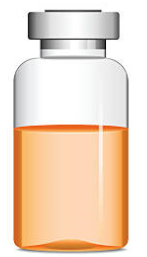

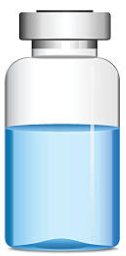

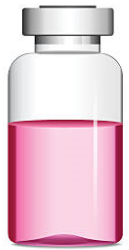


**Robot prepped natural standard**

**Labelled standard**

**Robot Blend**

**Analyst Blend**

**Ratio of 1**

**Ratio of 1**

*Figure S1. Schematic of the DEM-IDMS standard comparison process to compare automation and manual standard preparation*

Each sample blend was injected on the GC-MS/MS five times and quantified using the calibration blends injected immediately before and after the sample. The calculated mass fraction of amino acids in each of the “sample blends” was calculated using the reduced form of the DEM-IDMS equation (Equation S1).

*Equation S1: Simplified IDMS equation*

Where:

*W_x_* = the mass fraction of amino acids in the sample

*W_z_* = the mass fraction of the natural amino acids used to prepare the calibration blend

*m_z_* = mass of the natural amino acid solution added to the calibration blend

*m_x_* = mass of the sample added to the sample blend

*m_yc_* = mass of the labelled amino acid solution added to the calibration blend

*m_y_* = mass of the labelled amino acid solution added to the sample blend

*R’_B_* = measured ratio of the sample blend

*R’_BC_* = average measured ratio of the calibration blend injected before and after the sample

### Uncertainty calculation

The uncertainty associated with the final measured mass fraction was estimated by combining the relative standard uncertainties of the ratio measurements, the weighings, and the mass fraction of the amino acid solution (Equation S2).

*Equation S2: Uncertainty equation*

Where:

*U_Wz_* = the standard uncertainty associated with the mass fraction of the calibration solution

*w_z_* = the mass fraction of the calibration solution

*u_PR_* = the standard deviation of the ratios of R’B/R’Bc (n=5)

*P_R_* = the mean of R’B/R’Bc (n=5)

u*m_x_* = the uncertainty associated with the mass of sample used

*m_x_* = the mass of sample used

*um_y_* = the uncertainty associated with the mass of amino acid added to the sample

*m_y_* = the mass of labelled amino acid added to the sample

*um_z_* = the uncertainty associated with the mass of the amino acid added to the calibration blend

*m_z_* = the mass of amino acid added to the calibration blend

*um_yc_* = the uncertainty associated with the mass of labelled amino acid added to the calibration blend

*m_yc_* = the mass of labelled amino acid added to the calibration blend

The reported measurement uncertainty is either the root mean squared measurement uncertainty for the three independently prepared replicate samples or the standard deviation of the three measurements (whichever is larger) divided by the squareroot of *n* reported as an expanded uncertainty to the 95% confidence interval depending on the degrees of freedom.

If experiments were analysed over multiple batches, then between group and within group variation was also considered and added as part the uncertainty if required.

## Supplementary Information 2 – Mass fractions when compared to NIST SRM 2389a

Table S1. Certified Values for Amino Acids in SRM 2389a and the 10-fold dilution mass fraction aim for the robot preparations

|  | Certified Values for Amino Acids in SRM 2389a |  | Expanded uncertainty | Aim for mixed dilution on robot |
| --- | --- | --- | --- | --- |
|  | mg/g |  | mg/g | µg/g |
| Alanine | 0.223 | ± | 0.007 | 22.3 |
| Arginine | 0.436 | ± | 0.012 | 43.6 |
| Isoleucine | 0.32 | ± | 0.015 | 32 |
| Leucine | 0.319 | ± | 0.014 | 31.9 |
| Lysine | 0.353 | ± | 0.024 | 35.3 |
| Methionine | 0.373 | ± | 0.011 | 37.3 |
| Phenylalanine | 0.421 | ± | 0.014 | 42.1 |
| Proline | 0.282 | ± | 0.013 | 28.2 |
| Tyrosine | 0.459 | ± | 0.014 | 45.9 |
| Valine | 0.293 | ± | 0.012 | 29.3 |

## Supplementary Information 3 – Multiple reaction monitoring channels (MRMs)

*Table S2. MRM transitions*

| Compound | Precursor> product ion (*m/z*) | CE (eV) | Precursor> product ion (*m/z*) | CE (eV) | Dwell (ms) | Retention time (min) |
| --- | --- | --- | --- | --- | --- | --- |
| Alanine | 260>158 | 13 | 260>232 | 5 | 40 | 7.2 |
| Alanine* ^13^C_3_,^15^N | 264>161 | 13 | 264>235 | 5 | 40 | 7.2 |
| Valine | 288>260 | 13 | 288>186 | 7 | 40 | 8.5 |
| Valine* ^13^C_5_,^15^N | 294>265 | 13 | 294>191 | 7 | 40 | 8.5 |
| Leucine | 302>170 | 6 | 200>88 | 10 | 40 | 9 |
| Leucine* ^13^C_6_, ^15^N | 309>177 | 6 | 206>90 | 10 | 40 | 9 |
| Isoleucine | 302>274 | 6 | 302>170 | 4 | 40 | 9.4 |
| Isoleucine* ^13^C_6_ | 308>279 | 6 | 308>176 | 4 | 40 | 9.4 |
| Proline | 286>184 | 10 | 286>258 | 8 | 40 | 9.8 |
| Proline* ^13^C_5_,^15^N | 292>189 | 10 | 292>263 | 8 | 40 | 9.8 |
| Methionine | 292>147 | 7 | 292>244 | 6 | 40 | 11.9 |
| Methionine* Methyl-^13^C, D_3_ | 296>147 | 7 | 296>248 | 6 | 40 | 11.9 |
| Phenylalanine | 336>308 | 8 | 336>204 | 10 | 40 | 13.1 |
| Phenylalanine* ^13^C_9_, ^15^N | 346>317 | 8 | 346>214 | 10 | 40 | 13.1 |
| Lysine/HCl | 431>272 | 18 | 431>300 | 8 | 40 | 15.3 |
| Lysine/2HCl* ^13^C_6_,^15^N_2_ | 439>278 | 18 | 439>307 | 8 | 40 | 15.3 |
| Arginine | 442>169 | 18 | 442>199 | 17 | 40 | 15.8 |
| Arginine/HCl* ^13^C_6_ | 448>171 | 18 | 448>203 | 17 | 40 | 15.8 |
| Tyrosine | 466>438 | 20 | 438>147 | 20 | 40 | 16.3 |
| Tyrosine* ^13^C_9_,^15^N | 476>447 | 20 | 447>147 | 20 | 40 | 16.3 |

*Transition for labelled analyte

## Supplementary Information 4 – System checks

For the Agilent system, two 2 mL compatible vials were filled with sand, while for the Gerstel system, one 2 mL and one 20 mL compatible vial were used, also filled with sand. The homemade weights were calibrated by the UK National Weights and Measures Laboratory. Batch runs were conducted to measure these vials prior to using the robot, with the weights being monitored throughout. Additionally, the Sartorius balance on the Gerstel required regular internal calibrations, and the MPS rail itself needed a hard restart every week to prevent errors.

## Supplementary Information 5 – Lid-type evaporation experiments

*Figure S2. Percentage evaporation of water from 2 mL vials with differing lids (screw cap and crimp cap with and without slit) after a single pierced addition of water over 72 hours. The intercepts were tested by Student’s t-test and found to not be statistically different to zero.*

*Figure S3. Percentage evaporation of acetonitrile from 2 mL vials with differing lids (screw cap and crimp cap with and without slit) after a single pierced addition of acetonitrile over 72 hours. The intercepts were tested by Student’s t-test and found to not be statistically different to zero.*

Table S3. Linear regression and t-tests for the evaporation of water with different cap types

| **Water Percent loss:** | | | | | | | |
| --- | --- | --- | --- | --- | --- | --- | --- |
| **Crimp Lid without Slit** | | **Screw Lid without Slit** | | **Screw Lid Pre-Slit** | | **Crimp Lid Pre-Slit** | |
| **Slope** | 0.003 | **Slope** | 0.006 | **Slope** | 0.013 | **Slope** | 0.020 |
| **Std error** | 0.0003 | **Std error** | 0.0006 | **Std error** | 0.0034 | **Std error** | 0.0006 |
| **Intercept** | 0.02 | **Intercept** | 0.06 | **Intercept** | 0.33 | **Intercept** | 0.05 |
| **Std error** | 0.01 | **Std error** | 0.02 | **Std error** | 0.13 | **Std error** | 0.02 |
| **Crimp Pre-slit/Crimp no slit Slope t-test** | | **Screw Pre-slit/Screw no slit Slope t-test** | | **Screw Pre-slit/Crimp Pre-slit Slope t-test** | | **Screw no-slit/Crimp no-slit Slope t-test** | |
| **Bias** | -0.017 | **Bias** | -0.007 | **Bias** | -0.007 | **Bias** | -0.002 |
| **Standard error** | 0.001 | **Standard error** | 0.003 | **Standard error** | 0.003 | **Standard error** | 7.057E-04 |
| **t-value** | -24.292 | **t-value** | -1.977 | **t-value** | -2.075 | **t-value** | -3.472 |
| **Degrees of freedom** | 8 | **Degrees of freedom** | 8 | **Degrees of freedom** | 8 | **Degrees of freedom** | 8 |
| **p-value (based on T distribution)** | 8.799E-09 | **p-value (based on T distribution)** | 0.083 | **p-value (based on T distribution)** | 0.072 | **p-value (based on T distribution)** | 0.008 |

Table S4. Linear regression and t-tests for the evaporation of acetonitrile with different cap types

| **Acetonitrile Percent loss:** | | | | | | | |
| --- | --- | --- | --- | --- | --- | --- | --- |
| **Crimp Lid without Slit** | | **Screw Lid without Slit** | | **Screw Lid Pre-Slit** | | **Crimp Lid Pre-Slit** | |
| **Slope** | 0.07 | **Slope** | 0.08 | **Slope** | 0.23 | **Slope** | 0.27 |
| **Std error** | 0.001 | **Std error** | 0.005 | **Std error** | 0.009 | **Std error** | 0.004 |
| **Intercept** | 0.01 | **Intercept** | 0.46 | **Intercept** | 0.90 | **Intercept** | -0.35 |
| **Std error** | 0.03 | **Std error** | 0.18 | **Std error** | 0.35 | **Std error** | 0.15 |
| **Crimp Pre-slit/Crimp no slit Slope t-test** | | **Screw Pre-slit/Screw no slit Slope t-test** | | **Screw Pre-slit/Crimp Pre-slit Slope t-test** | | **Screw no-slit/Crimp no-slit Slope t-test** | |
| **Bias** | -0.192 | **Bias** | -0.151 | **Bias** | -0.034 | **Bias** | -0.007 |
| **Standard error** | 0.004 | **Standard error** | 0.011 | **Standard error** | 0.010 | **Standard error** | 0.005 |
| **t-value** | -46.896 | **t-value** | -14.180 | **t-value** | -3.365 | **t-value** | -1.432 |
| **Degrees of freedom** | 8 | **Degrees of freedom** | 8 | **Degrees of freedom** | 8 | **Degrees of freedom** | 8 |
| **p-value (based on T distribution)** | 4.725E-11 | **p-value (based on T distribution)** | 5.953E-07 | **p-value (based on T distribution)** | 0.010 | **p-value (based on T distribution)** | 0.190 |

## Supplementary Information 6 – Data and settings for accurate dispensing

Table S5. Example of the volume programmed for the robot to transfer, and the weight measured for consecutive water additions. For simplicity, all mass to volume calculations assumed water density of 1 g/cm3.

| Aim (µL) | Syringe | Weight (mg) | % of Target |
| --- | --- | --- | --- |
| 4098.4 | 5 mL | 4085.5 | 99.7 |
| 29.7 | 250 µL | 29.8 | 100.1 |
| 114.1 | 250 µL | 114.0 | 99.9 |
| 24.9 | 250 µL | 24.9 | 100.3 |
| 117.6 | 250 µL | 117.8 | 100.2 |
| 63.9 | 250 µL | 63.8 | 99.9 |
| 71.9 | 250 µL | 72.2 | 100.4 |
| 57.2 | 250 µL | 57.4 | 100.4 |
| 78.9 | 250 µL | 79.0 | 100.1 |
| 24.4 | 250 µL | 24.8 | 101.5 |
| 119.2 | 250 µL | 119.4 | 100.2 |
| 199.7 | 250 µL | 200.5 | 100.4 |

### Agilent WorkBench workflow and settings for accurate dispensing

1. Weigh vial x 3
2. Move vials to turret
3. Syringe wash solvent A (MeCN/H2O 50/50 v/v) x2,
4. Syringe wash solvent B (0.1M HCl) x2
5. Dispense some sample to waste (pierce septum)
6. Syringe pump (fill/dispense) within sample vial x2 (pierce septum)
7. Fill syringe with sample, overfill and airgap added (pierce septum)
8. Dispense syringe at destination vial
9. Dispense accurate add addition to waste
10. Syringe wash solvent A (MeCN/H2O 50/50 v/v) x2,
11. Syringe wash solvent B (0.1M HCl) x2
12. Weigh vial x 3

*Table S6. Syringe speed settings on Agilent Workbench*

| **Syringe Size (µL)** | **Fill Speed (µL/min)** | **Eject Speed (µL/min)** | **Airgap (% of syringe)** | **Overfill (% of syringe)** |
| --- | --- | --- | --- | --- |
| 100 | 100 | 2000 | 5 | 5 |
| 250 | 100 | 2000 | 5 | 5 |
| 500 | 500 | 1000 | 5 | 5 |

### Gerstel MPS workflow and settings for accurate dispensing

1. Weigh vial x 3
2. Syringe wash solvent A (MeCN/H2O 50/50 v/v) x2,
3. Syringe wash solvent B (0.1M HCl) x2
4. Move vials to decapper and decap
5. Syringe pump (fill/dispense) within sample x2
6. Fill syringe with sample, accurate addition added
7. Dispense syringe at destination vial
8. Dispense accurate add addition to waste
9. Recap vials
10. Syringe wash solvent A (MeCN/H2O 50/50 v/v) x2,
11. Syringe wash solvent B (0.1M HCl) x2
12. Weigh vial x 3

*Table S7. Syringe speed settings on Gerstel MPS*

| **Syringe Size (µL)** | **Fill Speed (µL/s)** | **Eject Speed (µL/s)** | **Post Add Delay (s)** | **Overfill (% of syringe)** |
| --- | --- | --- | --- | --- |
| 250 | 10 | 1000 | 2 | 10 |
| 1000 | 100 | 1000 | 5 | 10 |
| 5000 | 500 | 500 | 10 | 10 |

## Supplementary Information 7 – Student’s t-test information for Agilent and Gerstel comparison

Table S8. Student’s t-test data for Agilent and Gerstel comparison. Due to the difference in uncertainty between the gravimetric and the measured values the Welch-Satterthwaite equation was used to calculate the degrees of freedom and rounded down to the nearest integers.

| Agilent WB Blends | Mass Fraction (µg/g) | Uncertainty (µg/g) (k=1) |  |  | t-test |  |  |  |
| --- | --- | --- | --- | --- | --- | --- | --- | --- |
| Gravimetric value | 58.28 | 0.06 | **Standard deviation** | 0.62 | **Gravimetric value** | 58.28 | **Bias** | 0.08 |
| Agilent WB Blends 1 | 57.81 | 0.57 | **Root mean square uncertainty** | 0.52 | **Unexpanded uncertainty** | 0.06 | **Standard error** | 0.36 |
| Agilent WB Blends 2 | 59.03 | 0.52 | **Uncertainty of mean** | 0.36 | **Measured Value** | 58.36 | **t-value** | 0.21 |
| Agilent WB Blends 3 | 58.23 | 0.47 | **Expanded uncertainty** | 0.72 | **Unexpanded uncertainty** | 0.36 | **Degrees of freedom** | 2 |
| Measured average | 58.36 |  | **Percentage uncertainty** | 1.2 |  |  | **p-value (based on T distribution)** | 8.54E-01 |
| Agilent WB 2 Step Dil | **Mass Fraction (µg/g)** | **Uncertainty (µg/g) (k=1)** |  |  | **t-test** |  |  |  |
| Gravimetric value | 60.29 | 0.06 | **Standard deviation** | 0.22 | **Gravimetric value** | 60.29 | **Bias** | -1.96 |
| Agilent WB 2 Dil 1 | 58.52 | 0.43 | **Root mean square uncertainty** | 0.35 | **Unexpanded uncertainty** | 0.06 | **Standard error** | 0.21 |
| Agilent WB 2 Dil 2 | 58.09 | 0.26 | **Uncertainty of mean** | 0.20 | **Measured Value** | 58.33 | **t-value** | -9.22 |
| Agilent WB 2 Dil 3 | 58.37 | 0.35 | **Expanded uncertainty** | 0.41 | **Unexpanded uncertainty** | 0.20 | **Degrees of freedom** | 2 |
| Measured average | 58.33 |  | **Percentage uncertainty** | 0.7 |  |  | **p-value (based on T distribution)** | 1.16E-02 |
| Gerstel MPS Blends | **Mass Fraction (µg/g)** | **Uncertainty (µg/g) (k=1)** |  |  | **t-test** |  |  |  |
| Gravimetric value | 58.28 | 0.06 | **Standard deviation** | 0.01 | **Gravimetric value** | 58.28 | **Bias** | -2.29 |
| Gerstel MPS Blends 1 | 56.00 | 0.43 | **Root mean square uncertainty** | 0.35 | **Unexpanded uncertainty** | 0.06 | **Standard error** | 0.21 |
| Gerstel MPS Blends 2 | 56.00 | 0.26 | **Uncertainty of mean** | 0.20 | **Measured Value** | 55.99 | **t-value** | -10.74 |
| Gerstel MPS Blends 3 | 55.98 | 0.35 | **Expanded uncertainty** | 0.41 | **Unexpanded uncertainty** | 0.20 | **Degrees of freedom** | 2 |
| Measured average | 55.99 |  | **Percentage uncertainty** | 0.7 |  |  | **p-value (based on T distribution)** | 8.56E-03 |

| Gerstel MPS 1 Step Dil | Mass Fraction (µg/g) | Uncertainty (µg/g) (k=1) |  |  | t-test |  |  |  |
| --- | --- | --- | --- | --- | --- | --- | --- | --- |
| Gravimetric value | 57.29 | 0.06 | **Standard deviation** | 0.07 | **Gravimetric value** | 57.29 | **Bias** | -1.05 |
| Gerstel MPS 1 Dil 1 | 56.26 | 0.58 | **Root mean square uncertainty** | 0.55 | **Unexpanded uncertainty** | 0.06 | **Standard error** | 0.33 |
| Gerstel MPS 1 Dil 2 | 56.16 | 0.68 | **Uncertainty of mean** | 0.32 | **Measured Value** | 56.24 | **t-value** | -3.22 |
| Gerstel MPS 1 Dil 3 | 56.29 | 0.35 | **Expanded uncertainty** | 0.64 | **Unexpanded uncertainty** | 0.32 | **Degrees of freedom** | 2 |
| Measured average | 56.24 |  | **Percentage uncertainty** | 1.1 |  |  | **p-value (based on T distribution)** | 8.45E-02 |
| Gerstel MPS 2 Step Dil | **Mass Fraction (µg/g)** | **Uncertainty (µg/g) (k=1)** |  |  | **t-test** |  |  |  |
| Gravimetric value | 58.16 | 0.06 | **Standard deviation** | 0.42 | **Gravimetric value** | 58.16 | **Bias** | -1.04 |
| Gerstel MPS 2 Dil 1 | 56.65 | 0.22 | **Root mean square uncertainty** | 0.35 | **Unexpanded uncertainty** | 0.06 | **Standard error** | 0.25 |
| Gerstel MPS 2 Dil 2 | 57.46 | 0.27 | **Uncertainty of mean** | 0.24 | **Measured Value** | 57.12 | **t-value** | -4.19 |
| Gerstel MPS 2 Dil 3 | 57.25 | 0.50 | **Expanded uncertainty** | 0.48 | **Unexpanded uncertainty** | 0.24 | **Degrees of freedom** | 2 |
| Measured average | 57.12 |  | **Percentage uncertainty** | 0.8 |  |  | **p-value (based on T distribution)** | 5.26E-02 |

## Supplementary Information 8 – Manual Syringe Experiment Data

*Figure S4. Graph of Phenylalanine calculated mass fractions from each experiment replicate (blue) of manual syringe dilution through septa versus the average of the three (red) and the gravimetric value (pink) – error bars show ± the expanded uncertainty (k=2). The pink gravimetric value and red measured value should overlap uncertainties for a comparable experiment. The results through the septa are inconsistent. During the first replicate, a droplet was observed that remained on the syringe tip and was wiped through the septum. For the subsequent two replicates, the analyst attempted to displace the droplet prior to passing the syringe through the septum.* *The inconsistency across the set therefore reflects the sensitivity of the procedure to the droplet transfer rather than random variation, and this is why we concluded that the septa‑based approach introduced a systematic risk of bias.*

*Figure S5. Graph of Phenylalanine calculated mass fractions from each experiment replicate (blue) of manual syringe dilution without a septum versus the average of the three (red) and the gravimetric value (pink) – error bars show ± the expanded uncertainty (k=2). The pink gravimetric value and red measured value should overlap uncertainties for a comparable experiment. The results without the septa are consistent.*

Table S9. Student’s t-test results manual preparation through septa

| **Phenylalanine Manual Dilution through septa 1** | **Mass Fraction (µg/g)** | **Uncertainty (µg/g) (k=1)** |  |  |  | **t-test** |  |  |  |
| --- | --- | --- | --- | --- | --- | --- | --- | --- | --- |
| Gravimetric value | 60.12 | 0.06 | **Standard deviation** | 1.15 |  | **Gravimetric value** | 60.12 | **Bias** | -3.17 |
| Syringe Blends 1-1 | 56.18 | 0.58 | **Root mean square uncertainty** | 0.44 |  | **Unexpanded uncertainty** | 0.06 | **Standard error** | 0.67 |
| Syringe Blends 1-2 | 56.41 | 0.17 | **Uncertainty of mean** | 0.67 |  | **Measured Value** | 56.96 | **t-value** | -4.73 |
| Syringe Blends 1-3 | 58.29 | 0.46 | **Expanded uncertainty** | 1.33 |  | **Unexpanded uncertainty** | 0.67 | **Degrees of freedom** | 2 |
| Measured average | 56.96 |  | **Percentage uncertainty** | 2.3 |  |  |  | **p-value(based on T distribution)** | 4.19E-02 |
| **Phenylalanine Manual Dilution through septa 2** | **Mass Fraction (µg/g)** | **Uncertainty (µg/g) (k=1)** |  |  |  | **t-test** |  |  |  |
| Gravimetric value | 59.02 | 0.06 | **Standard deviation** | 0.36 |  | **Gravimetric value** | 59.02 | **Bias** | -0.43 |
| Syringe Blends 2-1 | 58.60 | 0.49 | **Root mean square uncertainty** | 0.57 |  | **Unexpanded uncertainty** | 0.06 | **Standard error** | 0.34 |
| Syringe Blends 2-2 | 58.94 | 0.79 | **Uncertainty of mean** | 0.33 |  | **Measured Value** | 58.59 | **t-value** | -1.28 |
| Syringe Blends 2-3 | 58.22 | 0.36 | **Expanded uncertainty** | 0.66 |  | **Unexpanded uncertainty** | 0.33 | **Degrees of freedom** | 2 |
| Measured average | 58.59 |  | **Percentage uncertainty** | 1.1 |  |  |  | **p-value(based on T distribution)** | 0.33 |
| **Phenylalanine Manual Dilution through septa 3** | **Mass Fraction (µg/g)** | **Uncertainty (µg/g) (k=1)** |  |  |  | **t-test** |  |  |  |
| Gravimetric value | 56.76 | 0.06 | **Standard deviation** | 0.55 |  | **Gravimetric value** | 56.76 | **Bias** | 0.29 |
| Syringe Blends 3-1 | 56.42 | 0.40 | **Root mean square uncertainty** | 0.43 |  | **Unexpanded uncertainty** | 0.06 | **Standard error** | 0.32 |
| Syringe Blends 3-2 | 57.26 | 0.56 | **Uncertainty of mean** | 0.32 |  | **Measured Value** | 57.05 | **t-value** | 0.90 |
| Syringe Blends 3-3 | 57.46 | 0.28 | **Expanded uncertainty** | 0.63 |  | **Unexpanded uncertainty** | 0.32 | **Degrees of freedom** | 2 |
| Measured average | 57.05 |  | **Percentage uncertainty** | 1.1 |  |  |  | **p-value(based on T distribution)** | 0.46 |

Table S10. t-test results manual preparation without septa

| **Phenylalanine Manual Dilution without septa 1** | **Mass Fraction (µg/g)** | **Uncertainty (µg/g) (k=1)** |  |  |  | **t-test** |  |  |  |
| --- | --- | --- | --- | --- | --- | --- | --- | --- | --- |
| Gravimetric value | 56.05 | 0.06 | **Standard deviation** | 0.35 |  | **Gravimetric value** | 56.05 | **Bias** | 0.15 |
| Syringe Blends 1-1 | 56.50 | 0.32 | **Root mean square uncertainty** | 0.47 |  | **Unexpanded uncertainty** | 0.06 | **Standard error** | 0.27 |
| Syringe Blends 1-2 | 56.29 | 0.52 | **Uncertainty of mean** | 0.27 |  | **Measured Value** | 56.20 | **t-value** | 0.56 |
| Syringe Blends 1-3 | 55.82 | 0.53 | **Expanded uncertainty** | 0.54 |  | **Unexpanded uncertainty** | 0.27 | **Degrees of freedom** | 2 |
| Measured average | 56.20 |  | **Percentage uncertainty** | 1.0 |  |  |  | **p-value(based on T distribution)** | 0.63 |
| **Phenylalanine Manual Dilution without septa 2** | **Mass Fraction (µg/g)** | **Uncertainty (µg/g) (k=1)** |  |  |  | **t-test** |  |  |  |
| Gravimetric value | 55.19 | 0.06 | **Standard deviation** | 0.75 |  | **Gravimetric value** | 55.19 | **Bias** | -0.43 |
| Syringe Blends 2-1 | 53.94 | 0.60 | **Root mean square uncertainty** | 0.50 |  | **Unexpanded uncertainty** | 0.06 | **Standard error** | 0.44 |
| Syringe Blends 2-2 | 54.94 | 0.56 | **Uncertainty of mean** | 0.43 |  | **Measured Value** | 54.76 | **t-value** | -0.98 |
| Syringe Blends 2-3 | 55.41 | 0.30 | **Expanded uncertainty** | 0.87 |  | **Unexpanded uncertainty** | 0.43 | **Degrees of freedom** | 2 |
| Measured average | 54.76 |  | **Percentage uncertainty** | 1.6 |  |  |  | **p-value(based on T distribution)** | 0.43 |
| **Phenylalanine Manual Dilution without septa 3** | **Mass Fraction (µg/g)** | **Uncertainty (µg/g) (k=1)** |  |  |  | **t-test** |  |  |  |
| Gravimetric value | 55.37 | 0.06 | **Standard deviation** | 0.15 |  | **Gravimetric value** | 55.37 | **Bias** | -0.08 |
| Syringe Blends 3-1 | 55.33 | 0.40 | **Root mean square uncertainty** | 0.28 |  | **Unexpanded uncertainty** | 0.06 | **Standard error** | 0.17 |
| Syringe Blends 3-2 | 55.13 | 0.09 | **Uncertainty of mean** | 0.16 |  | **Measured Value** | 55.30 | **t-value** | -0.45 |
| Syringe Blends 3-3 | 55.43 | 0.26 | **Expanded uncertainty** | 0.32 |  | **Unexpanded uncertainty** | 0.16 | **Degrees of freedom** | 2 |
| Measured average | 55.30 |  | **Percentage uncertainty** | 0.6 |  |  |  | **p-value(based on T distribution)** | 0.69 |

## Supplementary Information 9 – Water Evaporation Experiment Additional Data

Table S11. Cumulative difference data for water evaporation experiment for three methods

|  | **Cumulative difference after 9 events** | | |  |  |
| --- | --- | --- | --- | --- | --- |
|  | **Automated piercing** | **Automated decap/recap** | **Manual decap/recap** |  |  |
| **Total evaporation (mg)** | -0.98 | -0.60 | -0.32 |  |  |
| **Standard deviation** | 0.14 | 0.02 | 0.06 |  |  |
| **Standard uncertainty in mean** | 0.08 | 0.01 | 0.03 |  |  |
| **Degrees of freedom** | 2 | 2 | 2 |  |  |
| **Automated Decapping/Piercing t-test** | | **Automated Piercing/Manual Decapping t-test** | | **Automated Decapping/Manual Decapping t-test** | |
| **Bias** | -0.379 | **Bias** | -0.653 | **Bias** | -0.274 |
| **Standard error** | 0.080 | **Standard error** | 0.086 | **Standard error** | 0.034 |
| **t-value** | -4.761 | **t-value** | -7.618 | **t-value** | -8.014 |
| **Degrees of freedom** | 4 | **Degrees of freedom** | 4 | **Degrees of freedom** | 4 |
| **p-value (based on T distribution)** | 0.009 | **p-value (based on T distribution)** | 0.002 | **p-value (based on T distribution)** | 0.001 |

Table S12. Variance data within each event for water evaporation experiment for three methods. The step change after event 4 was ignored - the standard deviation of this group likely contains and mixture of systematic events and random variation. However, step changes contribute to variability. Therefore, the F test preformed here is approximate in nature but does provide an indication.

|  | **Variance within each event (n=3)** | | |  |  |  |  |  |
| --- | --- | --- | --- | --- | --- | --- | --- | --- |
|  | **Automated piercing** | **Automated decap/recap** | **Manual decap/recap** |  |  |  |  |  |
| **Event 2** | 0.002 | 0.001 | 0.001 |  |  |  |  |  |
| **Event 3** | 0.004 | 0.0002 | 0.006 |  |  |  |  |  |
| **Event 4** | 0.009 | 0.001 | 0.010 |  |  |  |  |  |
| **Event 5** | 0.048 | 0.0003 | 0.022 |  |  |  |  |  |
| **Event 6** | 0.030 | 0.001 | 0.004 |  |  |  |  |  |
| **Event 7** | 0.021 | 0.001 | 0.004 |  |  |  |  |  |
| **Event 8** | 0.021 | 0.004 | 0.017 |  |  |  |  |  |
| **Event 9** | 0.019 | 0.0002 | 0.003 |  |  |  |  |  |
| **Average** | 0.019 | 0.001 | 0.008 |  |  |  |  |  |
| **Standard deviation of trend of curve** | **0.138** | **0.031** | **0.092** |  |  |  |  |  |
| **Automated Decapping/Piercing variance f-test** | | | **Automated Piercing/Manual Decapping variance f-test** | | | **Automated Decapping/Manual Decapping variance f-test** | | |
|  | *0.002* | *0.001* |  | *0.001* | *0.002* |  | *0.001* | *0.001* |
| Mean | 0.022 | 0.001 | Mean | 0.010 | 0.022 | Mean | 0.010 | 0.001 |
| Variance | 0.0002 | 0.000002 | Variance | 0.0001 | 0.0002 | Variance | 0.00005 | 0.000002 |
| Observations | 7 | 7 | Observations | 7 | 7 | Observations | 7 | 7 |
| df | 6 | 6 | df | 6 | 6 | df | 6 | 6 |
| F | 120.1 |  | F | 0.2 |  | F | 29.7 |  |
| P(F<=f) one-tail | 5.6E-06 |  | P(F<=f) one-tail | 5.7E-02 |  | P(F<=f) one-tail | 3.3E-04 |  |
| F Critical one-tail | 4.3 |  | F Critical one-tail | 0.2 |  | F Critical one-tail | 4.3 |  |

## Supplementary Information 10 – Acetonitrile Evaporation Experiment Data


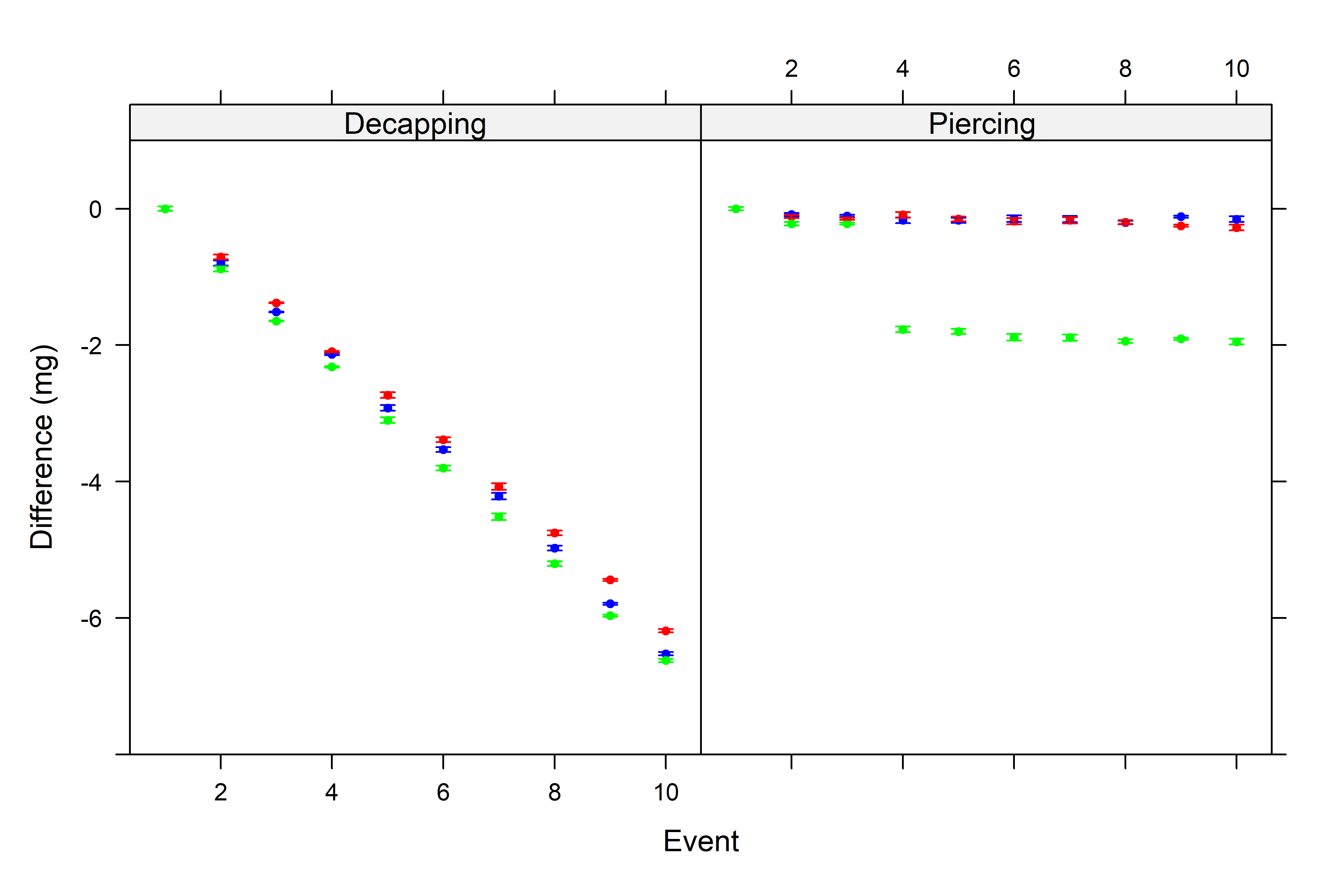


Figure S6. Comparison of evaporation over two methods –automated decapping and automated piercing. Each colour represents an analytical replicate. The cumulative loss of acetonitrile after each piercing or decapping event. Error bars are equal to +/- the standard deviation of weighing replicates (n=3). For simplicity, all mass to volume calculations assumed water density of 1 g/cm3.

Table S13. Cumulative difference data for acetonitrile evaporation experiment for two methods

|  | **Cumulative difference after 9 events in acetonitrile** | |
| --- | --- | --- |
|  | **Automated piercing** | **Automated decap/recap** |
| **Total evaporation (mg)** | -0.758 | -5.732 |
| **Standard deviation** | 0.9972 | 0.2680 |
| **Standard uncertainty in mean** | 0.5757 | 0.1548 |
| **Degrees of freedom** | 2 | 2 |
| **Automated Decapping/Piercing t-test** | |  |
| **Bias** | 4.974 |  |
| **Standard error** | 0.596 |  |
| **t-value** | 8.344 |  |
| **Degrees of freedom** | 4 |  |
| **p-value (based on T distribution)** | 0.001 |  |

Table S14. Variance data within each event for acetonitrile evaporation experiment for two methods. The step change after event 3 was ignored - the standard deviation of this group likely contains and mixture of systematic events and random variation. However, step changes contribute to variability. Therefore, the F test preformed here is approximate in nature but does provide an indication.

|  | **Variance within each event (n=3)** | |
| --- | --- | --- |
|  | **Automated piercing** | **Automated decap/recap** |
| **Event 2** | 0.005 | 0.008 |
| **Event 3** | 0.003 | 0.018 |
| **Event 4** | 0.898 | 0.014 |
| **Event 5** | 0.897 | 0.034 |
| **Event 6** | 0.985 | 0.045 |
| **Event 7** | 0.996 | 0.051 |
| **Event 8** | 1.009 | 0.051 |
| **Event 9** | 0.994 | 0.072 |
| **Average** | 0.723 | 0.037 |
| **Standard deviation of trend of curve** | **0.851** | **0.191** |
| **Automated Decapping/Piercing variance f-test** | | |
|  | *0.005* | *0.008* |
| Mean | 0.826 | 0.041 |
| Variance | 0.1338 | 0.0004 |
| Observations | 7 | 7 |
| df | 6 | 6 |
| F | 325.1 |  |
| P(F<=f) one-tail | 2.9E-07 |  |
| F Critical one-tail | 4.3 |  |

## Supplementary Information 11 – Linear regression for evaporation from different volumes data

Figure S7. Log transformation and linearisation of the absolute evaporation data from three different volumes in a 10 mL vial

Table S15. t-tests performed on slopes and intercepts to assess significant differences between absolute loss from three volumes in a 10 mL vial

| **Total loss:** | | | | | |
| --- | --- | --- | --- | --- | --- |
| **Linear regression on 100 mg group** | | **Linear regression on 500 mg group** | | **Linear regression on 2000 mg group** | |
| **Slope** | 0.68 | **Slope** | 0.67 | **Slope** | 0.67 |
| **Std error** | 0.01 | **Std error** | 0.02 | **Std error** | 0.02 |
| **Intercept** | -0.88 | **Intercept** | -0.77 | **Intercept** | -0.75 |
| **Std error** | 0.02 | **Std error** | 0.03 | **Std error** | 0.03 |
| **Slope 100/500 t-test** | | **Slope 500/2000 t-test** | | **Slope 2000/100 t-test** | |
| **Bias** | 0.014 | **Bias** | -0.004 | **Bias** | -0.010 |
| **Standard error** | 0.024 | **Standard error** | 0.026 | **Standard error** | 0.023 |
| **t-value** | 0.601 | **t-value** | -0.163 | **t-value** | -0.426 |
| **Degrees of freedom** | 14 | **Degrees of freedom** | 14 | **Degrees of freedom** | 14 |
| **p-value (based on T distribution)** | 0.557 | **p-value (based on T distribution)** | 0.873 | **p-value (based on T distribution)** | 0.677 |
| **Intercept 100/500 t-test** | | **Intercept 500/2000 t-test** | | **Intercept 2000/100 t-test** | |
| **Bias** | -0.106 | **Bias** | -0.021 | **Bias** | 0.127 |
| **Standard error** | 0.036 | **Standard error** | 0.040 | **Standard error** | 0.036 |
| **t-value** | -2.946 | **t-value** | -0.520 | **t-value** | 3.575 |
| **Degrees of freedom** | 14 | **Degrees of freedom** | 14 | **Degrees of freedom** | 14 |
| **p-value (based on T distribution)** | 1.06E-02 | **p-value (based on T distribution)** | 0.611 | **p-value (based on T distribution)** | 3.04E-03 |

Figure S8. Log transformation and linearisation of the percentage evaporation data from three different volumes in a 10 mL vial

Table S16. t-tests performed on slopes and intercepts to assess significant differences between percentage loss from three volumes in a 10 mL vial

| **Percent loss:** | | | | | |
| --- | --- | --- | --- | --- | --- |
| **Linear regression on 100 mg group** | | **Linear regression on 500 mg group** | | **Linear regression on 2000 mg group** | |
| **Slope** | 0.68 | **Slope** | 0.67 | **Slope** | 0.67 |
| **Std error** | 0.01 | **Std error** | 0.02 | **Std error** | 0.02 |
| **Intercept** | -0.88 | **Intercept** | -1.47 | **Intercept** | -2.05 |
| **Std error** | 0.02 | **Std error** | 0.03 | **Std error** | 0.03 |
| **Slope 100/500 t-test** | | **Slope 500/2000 t-test** | | **Slope 2000/100 t-test** | |
| **Bias** | 0.014 | **Bias** | -0.004 | **Bias** | -0.010 |
| **Standard error** | 0.024 | **Standard error** | 0.026 | **Standard error** | 0.023 |
| **t-value** | 0.601 | **t-value** | -0.163 | **t-value** | -0.426 |
| **Degrees of freedom** | 14 | **Degrees of freedom** | 14 | **Degrees of freedom** | 14 |
| **p-value (based on T distribution)** | 0.557 | **p-value (based on T distribution)** | 0.873 | **p-value (based on T distribution)** | 0.677 |
| **Intercept 100/500 t-test** | | **Intercept 500/2000 t-test** | | **Intercept 2000/100 t-test** | |
| **Bias** | 0.593 | **Bias** | 0.581 | **Bias** | -1.174 |
| **Standard error** | 0.036 | **Standard error** | 0.040 | **Standard error** | 0.036 |
| **t-value** | 16.395 | **t-value** | 14.500 | **t-value** | -32.966 |
| **Degrees of freedom** | 14 | **Degrees of freedom** | 14 | **Degrees of freedom** | 14 |
| **p-value (based on T distribution)** | 1.56E-10 | **p-value (based on T distribution)** | 7.97E-10 | **p-value (based on T distribution)** | 1.13E-14 |

## Supplementary Information 12 – Comparison with NIST material by decapping

Table S17. Student’s t-tests between gravimetric value of the NIST material and the measured value prepared by automation via decapping. Due to the difference in uncertainty between the certified and the measured values the Welch-Satterthwaite equation was used to calculate the degrees of freedom and rounded down to the nearest integers.

|  | **Student's t-tests between gravimetric value of the NIST material and the measured value prepared by automation *via* decapping** | | | |
| --- | --- | --- | --- | --- |
| **Leucine** | **Certified Value (µg/g)** | 31.81 | **Bias** | 1.07 |
|  | **Unexpanded uncertainty (µg/g)** | 0.70 | **Standard error** | 0.72 |
|  | **Percent expanded uncertainty** | 4.39 | **t-value** | 1.48 |
|  | **Measured Value (µg/g)** | 32.88 | **Degrees of freedom** | 290 |
|  | **Unexpanded uncertainty (µg/g)** | 0.17 | **p-value (based on T distribution)** | 0.14 |
|  | **Percent expanded uncertainty** | 1.06 |  |  |
|  | **Percent difference** | 3.35 |  |  |
| **Alanine** | **Gravimetric Value (µg/g)** | 22.24 | **Bias** | 0.08 |
|  | **Unexpanded uncertainty (µg/g)** | 0.35 | **Standard error** | 0.38 |
|  | **Percent expanded uncertainty** | 3.20 | **t-value** | 0.22 |
|  | **Measured Value (µg/g)** | 22.32 | **Degrees of freedom** | 32 |
|  | **Unexpanded uncertainty (µg/g)** | 0.16 | **p-value (based on T distribution)** | 0.83 |
|  | **Percent expanded uncertainty** | 1.47 |  |  |
|  | **Percent difference** | 0.38 |  |  |
| **Valine** | **Gravimetric Value (µg/g)** | 29.22 | **Bias** | 0.08 |
|  | **Unexpanded uncertainty (µg/g)** | 0.60 | **Standard error** | 0.63 |
|  | **Percent expanded uncertainty** | 4.09 | **t-value** | 0.13 |
|  | **Measured Value (µg/g)** | 29.30 | **Degrees of freedom** | 124 |
|  | **Unexpanded uncertainty (µg/g)** | 0.19 | **p-value (based on T distribution)** | 0.90 |
|  | **Percent expanded uncertainty** | 1.28 |  |  |
|  | **Percent difference** | 0.28 |  |  |
| **Lysine** | **Gravimetric Value (µg/g)** | 35.21 | **Bias** | 0.89 |
|  | **Unexpanded uncertainty (µg/g)** | 1.20 | **Standard error** | 1.21 |
|  | **Percent expanded uncertainty** | 6.80 | **t-value** | 0.74 |
|  | **Measured Value (µg/g)** | 36.10 | **Degrees of freedom** | 3903 |
|  | **Unexpanded uncertainty (µg/g)** | 0.15 | **p-value (based on T distribution)** | 0.46 |
|  | **Percent expanded uncertainty** | 0.85 |  |  |
|  | **Percent difference** | 2.53 |  |  |
| **Isoleucine** | **Gravimetric Value (µg/g)** | 31.91 | **Bias** | 1.12 |
|  | **Unexpanded uncertainty (µg/g)** | 0.75 | **Standard error** | 0.76 |
|  | **Percent expanded uncertainty** | 4.69 | **t-value** | 1.48 |
|  | **Measured Value (µg/g)** | 33.04 | **Degrees of freedom** | 40101 |
|  | **Unexpanded uncertainty (µg/g)** | 0.11 | **p-value (based on T distribution)** | 0.14 |
|  | **Percent expanded uncertainty** | 0.65 |  |  |
|  | **Percent difference** | 3.51 |  |  |
| **Proline** | **Gravimetric Value (µg/g)** | 28.12 | **Bias** | 0.66 |
|  | **Unexpanded uncertainty (µg/g)** | 0.65 | **Standard error** | 0.76 |
|  | **Percent expanded uncertainty** | 5.02 | **t-value** | 0.86 |
|  | **Measured Value (µg/g)** | 28.78 | **Degrees of freedom** | 12 |
|  | **Unexpanded uncertainty (µg/g)** | 0.40 | **p-value (based on T distribution)** | 0.41 |
|  | **Percent expanded uncertainty** | 3.05 |  |  |
|  | **Percent difference** | 2.34 |  |  |
| **Arginine** | **Gravimetric Value (µg/g)** | 43.48 | **Bias** | 0.13 |
|  | **Unexpanded uncertainty (µg/g)** | 0.60 | **Standard error** | 0.61 |
|  | **Percent expanded uncertainty** | 2.75 | **t-value** | 0.21 |
|  | **Measured Value (µg/g)** | 43.61 | **Degrees of freedom** | 404 |
|  | **Unexpanded uncertainty (µg/g)** | 0.14 | **p-value (based on T distribution)** | 0.83 |
|  | **Percent expanded uncertainty** | 0.62 |  |  |
|  | **Percent difference** | 0.30 |  |  |
| **Phenylalanine** | **Gravimetric Value (µg/g)** | 41.99 | **Bias** | -0.64 |
|  | **Unexpanded uncertainty (µg/g)** | 0.70 | **Standard error** | 0.79 |
|  | **Percent expanded uncertainty** | 3.46 | **t-value** | -0.81 |
|  | **Measured Value (µg/g)** | 41.35 | **Degrees of freedom** | 21 |
|  | **Unexpanded uncertainty (µg/g)** | 0.36 | **p-value (based on T distribution)** | 0.43 |
|  | **Percent expanded uncertainty** | 1.83 |  |  |
|  | **Percent difference** | -1.52 |  |  |
| **Methionine** | **Gravimetric Value (µg/g)** | 37.20 | **Bias** | 0.09 |
|  | **Unexpanded uncertainty (µg/g)** | 0.55 | **Standard error** | 0.57 |
|  | **Percent expanded uncertainty** | 2.95 | **t-value** | 0.16 |
|  | **Measured Value (µg/g)** | 37.29 | **Degrees of freedom** | 191 |
|  | **Unexpanded uncertainty (µg/g)** | 0.15 | **p-value (based on T distribution)** | 0.87 |
|  | **Percent expanded uncertainty** | 0.82 |  |  |
|  | **Percent difference** | 0.25 |  |  |
| **Tyrosine** | **Gravimetric Value (µg/g)** | 45.78 | **Bias** | -0.06 |
|  | **Unexpanded uncertainty (µg/g)** | 0.70 | **Standard error** | 1.04 |
|  | **Percent expanded uncertainty** | 4.85 | **t-value** | -0.06 |
|  | **Measured Value (µg/g)** | 45.72 | **Degrees of freedom** | 3 |
|  | **Unexpanded uncertainty (µg/g)** | 0.77 | **p-value (based on T distribution)** | 0.96 |
|  | **Percent expanded uncertainty** | 5.35 |  |  |
|  | **Percent difference** | -0.13 |  |  |

## Supplementary Information 13 – Comparison with NIST material by piercing

*Figure S9. The certified value of the NIST material (red) is compared to the measured value prepared by using the robotic liquid handling (blue). The black error bars are equal to +/- the expanded uncertainty of the uncertainty of the difference (k=95% confidence interval based on degrees of freedom). The robot prepared measured value (blue) are biased when compared to the certified value for the majority of analytes up to 7% where the measured value is more concentrated than the certified value.*

Table S18. Student’s t-tests between gravimetric value of the NIST material and the measured value prepared by automation via piercing septa.

|  | **Student t-tests between gravimetric value of the NIST material and the measured value prepared by automation *via* decapping** | | | |
| --- | --- | --- | --- | --- |
| **Leucine** | **Certified Value (µg/g)** | 31.81 | **Bias** | 1.86 |
|  | **Unexpanded uncertainty (µg/g)** | 0.70 | **Standard error** | 0.71 |
|  | **Percent expanded uncertainty** | 4.39 | **t-value** | 2.62 |
|  | **Measured Value (µg/g)** | 33.67 |  |  |
|  | **Unexpanded uncertainty (µg/g)** | 0.12 | **p-value (based on normal distribution)** | 0.01 |
|  | **Percent expanded uncertainty** | 0.72 |  |  |
|  | **Percent difference** | 5.84 |  |  |
| **Alanine** | **Gravimetric Value (µg/g)** | 22.24 | **Bias** | 0.88 |
|  | **Unexpanded uncertainty (µg/g)** | 0.35 | **Standard error** | 0.35 |
|  | **Percent expanded uncertainty** | 3.14 | **t-value** | 2.51 |
|  | **Measured Value (µg/g)** | 23.12 |  |  |
|  | **Unexpanded uncertainty (µg/g)** | 0.03 | **p-value (based on normal distribution)** | 0.02 |
|  | **Percent expanded uncertainty** | 0.26 |  |  |
|  | **Percent difference** | 3.95 |  |  |
| **Valine** | **Gravimetric Value (µg/g)** | 29.22 | **Bias** | 0.72 |
|  | **Unexpanded uncertainty (µg/g)** | 0.60 | **Standard error** | 0.60 |
|  | **Percent expanded uncertainty** | 4.09 | **t-value** | 1.20 |
|  | **Measured Value (µg/g)** | 29.94 |  |  |
|  | **Unexpanded uncertainty (µg/g)** | 0.08 | **p-value (based on normal distribution)** | 0.20 |
|  | **Percent expanded uncertainty** | 0.51 |  |  |
|  | **Percent difference** | 2.47 |  |  |
| **Lysine** | **Gravimetric Value (µg/g)** | 35.21 | **Bias** | 1.87 |
|  | **Unexpanded uncertainty (µg/g)** | 1.20 | **Standard error** | 1.20 |
|  | **Percent expanded uncertainty** | 6.80 | **t-value** | 1.56 |
|  | **Measured Value (µg/g)** | 37.08 |  |  |
|  | **Unexpanded uncertainty (µg/g)** | 0.12 | **p-value (based on normal distribution)** | 0.12 |
|  | **Percent expanded uncertainty** | 0.66 |  |  |
|  | **Percent difference** | 5.32 |  |  |
| **Isoleucine** | **Gravimetric Value (µg/g)** | 31.91 | **Bias** | 2.22 |
|  | **Unexpanded uncertainty (µg/g)** | 0.75 | **Standard error** | 0.75 |
|  | **Percent expanded uncertainty** | 4.69 | **t-value** | 2.95 |
|  | **Measured Value (µg/g)** | 34.13 |  |  |
|  | **Unexpanded uncertainty (µg/g)** | 0.08 | **p-value (based on normal distribution)** | 0.01 |
|  | **Percent expanded uncertainty** | 0.49 |  |  |
|  | **Percent difference** | 6.95 |  |  |
| **Proline** | **Gravimetric Value (µg/g)** | 28.12 | **Bias** | 1.68 |
|  | **Unexpanded uncertainty (µg/g)** | 0.65 | **Standard error** | 0.65 |
|  | **Percent expanded uncertainty** | 4.61 | **t-value** | 2.58 |
|  | **Measured Value (µg/g)** | 29.80 |  |  |
|  | **Unexpanded uncertainty (µg/g)** | 0.06 | **p-value (based on normal distribution)** | 0.01 |
|  | **Percent expanded uncertainty** | 0.38 |  |  |
|  | **Percent difference** | 5.97 |  |  |
| **Arginine** | **Gravimetric Value (µg/g)** | 43.48 | **Bias** | 1.15 |
|  | **Unexpanded uncertainty (µg/g)** | 0.60 | **Standard error** | 0.66 |
|  | **Percent expanded uncertainty** | 2.75 | **t-value** | 1.75 |
|  | **Measured Value (µg/g)** | 44.63 |  |  |
|  | **Unexpanded uncertainty (µg/g)** | 0.27 | **p-value (based on normal distribution)** | 0.09 |
|  | **Percent expanded uncertainty** | 1.22 |  |  |
|  | **Percent difference** | 2.64 |  |  |
| **Phenylalanine** | **Gravimetric Value (µg/g)** | 41.99 | **Bias** | 0.54 |
|  | **Unexpanded uncertainty (µg/g)** | 0.70 | **Standard error** | 0.71 |
|  | **Percent expanded uncertainty** | 3.32 | **t-value** | 0.77 |
|  | **Measured Value (µg/g)** | 42.53 | **Degrees of freedom** | 2.00 |
|  | **Unexpanded uncertainty (µg/g)** | 0.11 | **p-value (based on normal distribution)** | 0.30 |
|  | **Percent expanded uncertainty** | 0.54 |  |  |
|  | **Percent difference** | 1.29 |  |  |
| **Methionine** | **Gravimetric Value (µg/g)** | 37.20 | **Bias** | 1.45 |
|  | **Unexpanded uncertainty (µg/g)** | 0.55 | **Standard error** | 0.55 |
|  | **Percent expanded uncertainty** | 2.95 | **t-value** | 2.62 |
|  | **Measured Value (µg/g)** | 38.65 |  |  |
|  | **Unexpanded uncertainty (µg/g)** | 0.06 | **p-value (based on normal distribution)** | 0.01 |
|  | **Percent expanded uncertainty** | 0.29 |  |  |
|  | **Percent difference** | 3.89 |  |  |
| **Tyrosine** | **Gravimetric Value (µg/g)** | 45.78 | **Bias** | 2.29 |
|  | **Unexpanded uncertainty (µg/g)** | 0.70 | **Standard error** | 0.71 |
|  | **Percent expanded uncertainty** | 3.05 | **t-value** | 3.22 |
|  | **Measured Value (µg/g)** | 48.07 |  |  |
|  | **Unexpanded uncertainty (µg/g)** | 0.13 | **p-value (based on normal distribution)** | 0.002 |
|  | **Percent expanded uncertainty** | 0.56 |  |  |
|  | **Percent difference** | 5.00 |  |  |
